# Impedimetric Analysis of the Photocatalysis-Assisted Response of Patterned TiO_2_|ITO Electrodes Exposed to Artificial Sweat

**DOI:** 10.3390/s26082365

**Published:** 2026-04-11

**Authors:** Bozhidar I. Stefanov, Valentin M. Mateev, Boriana R. Tzaneva, Ivo T. Iliev

**Affiliations:** 1 Department of Chemistry, Faculty of Electronic Engineering and Technology, Technical University of Sofia, 8, Kliment Ohridski Blvd, 1000 Sofia, Bulgaria; borianatz@tu-sofia.bg; 2 Department of Electrical Apparatus, Faculty of Electrical Engineering, Technical University of Sofia, 8, Kliment Ohridski Blvd, 1000 Sofia, Bulgaria; vmateev@tu-sofia.bg; 3 Department of Electronics, Faculty of Electronic Engineering and Technology, Technical University of Sofia, 8, Kliment Ohridski Blvd, 1000 Sofia, Bulgaria; izi@tu-sofia.bg

**Keywords:** artificial sweat, TiO_2_, ITO, thin films, electrochemical impedance spectroscopy, lactic acid, urea, impedimetric response

## Abstract

We report the fabrication and electrochemical characterization of TiO_2_-based impedimetric sensors for the analysis of artificial sweat compositions. Two-electrode topologies were patterned on indium tin oxide (ITO) substrates: an interdigitated electrode (IDE) configuration and a Hilbert fractal electrode (HFE) geometry. TiO_2_ thin films with thickness up to 350 nm were deposited by dip-coating and evaluated as photoactive sensing layers. The impedimetric response of the sensors was investigated by electrochemical impedance spectroscopy in artificial sweat with composition varied in terms of ionic content (0–100 mM Na^+^) and organic content (2.5–30 mM lactic acid and 5–50 mM urea). Regardless of TiO_2_ thickness, the high-frequency response is predominantly governed by electrode topology, with the HFE design exhibiting up to 2.5-fold higher modulation compared to the IDE configuration. Under UV illumination, a low-frequency, photo-assisted response emerges, influenced by the TiO_2_ layer thickness and primarily sensitive to the organic components of the solution, particularly lactic acid. These results suggest that frequency-resolved impedance measurements in TiO_2_|ITO structures may enable partial differentiation between ionic conductivity and organic contributions in sweat, providing a promising basis for multi-parameter sweat analysis.

## 1. Introduction

Sweat composition analysis allows the monitoring of physiological parameters and metabolic state without the need for invasive sampling procedures [1,2,3]. However, conventional sweat sampling followed by ex situ analysis presents practical limitations that restrict its use in applications such as sports medicine, health monitoring, and personalized diagnostics [4,5]. Consequently, increasing interest has been directed toward the development of integrated sensors capable of continuously monitoring sweat composition in wearable devices [6,7,8,9].

Human sweat is composed primarily of water, electrolytes, and low-molecular-weight organic metabolites [2,10]. The dominant inorganic constituents are sodium (Na^+^, 10–90 mM), potassium (K^+^, 2–8 mM), and ammonia (NH_3_/NH_4_^+^, 1–8 mM), typically present with chloride (Cl^−^) as the main counterion [4,11,12]. In addition to electrolytes, sweat contains an organic fraction mainly represented by lactate (5–40 mM) and urea (4–12 mM), together with smaller amounts of glucose, amino acids, proteins, and other metabolites [4,11]. Sweat composition varies with physiological condition and the intensity of physical activity [13]. Among the measurable components, Na^+^ concentration is strongly associated with hydration status and sweating rate. Lactate represents one of the major organic constituents of sweat and is frequently monitored during physical exertion as an indicator related to metabolic activity [14,15]. Urea is another relevant metabolite originating from nitrogen metabolism and present in measurable concentrations in sweat [15]. Consequently, the simultaneous monitoring of electrolytes and selected organic metabolites in sweat can provide useful information about physiological processes occurring during physical activity.

Electrolyte concentrations in sweat can be monitored using conductometric or impedance-based sensors, since the overall electrical conductivity of sweat is largely determined by its ionic content. Typical sweat conductivity values range approximately between 2 and 11 mS cm^−1^ [16], and can be directly related to Na^+^/Cl^−^ concentration and overall electrolyte composition [12,17,18,19]. Multiple examples of wearable sensing platforms have been reported for real-time monitoring of sweat conductivity. Liu et al. demonstrated a simple conductometer based on parallel wire electrodes embedded in a PDMS mold, measuring sweat impedance at 100 kHz [20]. Steijlen et al. proposed a wearable sweat collection patch with integrated parallel electrodes enabling both conductivity and sweat rate monitoring via impedance measurements at 80 kHz, followed by off-line analysis [21]. Yuan employed an interdigitated electrode (IDE) array to measure impedance at 1 kHz, while sweat rate was determined using a microfluidic serpentine channel progressively covering the IDE surface [22]. Es Sebar et al. proposed an IDE-based electrode configuration for hydration monitoring via impedimetric measurements [23].

The performance of impedimetric sensors is strongly influenced by electrode geometry. Higher-density electrode topologies, such as interdigitated electrodes (IDEs), provide improved sensitivity compared to simple parallel electrodes due to their longer effective electrode path per unit area. However, IDE configurations can exhibit a nonlinear response because of the distribution of the electric field and the effective ionic current paths in the electrolyte [24,25]. IDE structures are nevertheless widely used in impedimetric sensor designs due to their simplicity and well-defined response, as well as the possibility to tune their electrical characteristics by adjusting parameters such as finger width and interelectrode spacing [26,27].

Recently, alternative electrode topologies based on fractal geometries have attracted increasing attention. Designs derived from the Hilbert space-filling curve exhibit high perimeter density and a more uniform electric field distribution, making them attractive for applications requiring large effective electrode interfaces. Such geometries have been explored extensively in energy storage devices [28,29] and have also been investigated in biomedical technologies such as neurostimulation systems [30]. More recently, fractal electrode architectures have been applied in sensing applications including chemoresistive VOC sensors [31,32], wearable strain sensors [33], and impedimetric biosensors [34].

In contrast to the impedimetric sensing of electrolyte content, the detection of organic metabolites in sweat is more challenging because it requires selective electrochemical reactions at the sensor surface. This selectivity is most commonly achieved using enzymatic detection schemes, in which enzymes such as lactate oxidase or urease catalyze reactions that generate measurable electrochemical signals. These signals typically appear as electrocatalytic currents or potential changes and are detected using techniques such as chronoamperometry [35,36,37]. Selectivity in impedance-based sensors can also be introduced by immobilizing specific antibodies on the electrode surface, which enables the detection of non-electroactive biomarkers such as cortisol [38,39,40].

Although enzymatic sensors provide high selectivity, their practical implementation in wearable devices can be limited by enzyme instability, restricted operational lifetime, and sensitivity to environmental conditions [36,41]. These limitations have motivated the development of non-enzymatic sensing approaches based on noble metals, transition metal oxides, and two-dimensional materials [8]. Such systems generally exhibit higher chemical stability and improved resistance to poisoning, although their selectivity toward specific metabolites is often lower than that of enzyme-based sensors [42,43,44]. Nevertheless, their robustness makes non-enzymatic materials attractive for long-term sensing applications in chemically aggressive environments such as sweat.

Examples of non-enzymatic sensors include an Au-decorated CuO chronoamperometric lactate sensor proposed by Arivazhagan et al., operating in the 0.1–88 µM range [45]. Kim et al. demonstrated an NiO-based lactic acid sensor operating between 0.6 and 35 mM [46], while de Oliveira et al. reported a Ni-based sensor with a linear range of 0.085–3.10 mM [47]. Chen et al. developed a wearable non-enzymatic glucose sensor based on PEDOT:PSS incorporating Au nanoparticles and aminated multi-walled carbon nanotubes, operating in the 50–600 µM range [48].

Among the materials explored for electrochemical sensing applications, titanium dioxide (TiO_2_) has attracted considerable interest due to its high chemical stability, non-toxicity, and corrosion resistance, which are desirable properties for sensors operating in contact with biological fluids [49]. TiO_2_ has been widely employed as a support material in both enzymatic and non-enzymatic electrochemical sensors [50]. Owing to the ease of immobilizing biomolecules such as enzymes and antibodies on TiO_2_ surfaces, it has been used in various biosensing platforms, including lactate detection reported by Casero et al. [51] and cortisol sensing demonstrated by Madhu et al. [40].

More recently, non-enzymatic TiO_2_-based sensing systems have also been explored. Examples include the amperometric glucose sensor based on reduced TiO_2−x_ thin films proposed by Villamizar et al. [52], dopamine sensors based on {001}-oriented TiO_2_ reported by Zhang et al. [53], and a wearable TiO_2_/carbon cloth composite sensor for L-cysteine detection demonstrated by Chen et al. [54].

In addition to its chemical stability, TiO_2_ is a well-known photocatalyst capable of generating electron–hole pairs under ultraviolet illumination [55,56]. These photogenerated charge carriers can increase the electrical conductivity of the material and participate in oxidation reactions of adsorbed organic species [57], enabling photo-assisted electrochemical detection mechanisms. Despite extensive research on TiO_2_-based sensing systems, however, the use of UV-activated TiO_2_ layers for the combined electrochemical sensing of electrolytes and organic metabolites remains largely unexplored.

A limited number of studies have demonstrated photoelectrochemical sensing based on TiO_2_ structures. For example, Su et al. reported glucose detection using pristine and GO_x_-modified TiO_2_ nanorods on FTO substrates under 380 nm illumination [58]. Similarly, Ke et al. demonstrated photoelectrochemical glucose detection in sweat using CuO-decorated TiO_2_ nanotubes under simulated solar irradiation (AM 1.5G) [59]. Furthermore, the photocatalytic and electrochemical responses of TiO_2_ films are known to depend strongly on film thickness and morphology, parameters that are rarely systematically addressed in the design of TiO_2_-based sweat sensors [60].

In this work, we investigate the influence of electrode topology and TiO_2_ layer thickness on the electrochemical response of TiO_2_ layers deposited onto patterned ITO readout electrodes in artificial sweat. Two-electrode configurations are compared: a conventional interdigitated electrode (IDE) array and a Hilbert fractal electrode (HFE) geometry. While similar comparisons of electrode topology have been reported for VOC sensing [31,61], and the advantages of HFE structures have been demonstrated in biosensing applications [34], direct comparisons for sweat conductivity monitoring remain lacking. In parallel, TiO_2_-based sensors and photoelectrochemical approaches have been explored for the detection of specific metabolites, predominantly glucose [58,59], typically employing nanotubular or catalyst-modified TiO_2_ architectures. However, systematic studies addressing the influence of TiO_2_ layer parameters on photoelectrochemical response are still limited. Therefore, in the present work, the TiO_2_ overlayer thickness is systematically varied to evaluate its influence on both electrolyte-dependent conductivity and photocatalytically assisted electrochemical processes. The sensors were studied using synthetic sweat solutions with controlled concentrations of Na^+^, lactic acid (LA), and urea, enabling evaluation of responses toward both ionic and organic components. Electrochemical impedance spectroscopy was employed to analyze sensing behavior and to assess the potential of TiO_2_-based photoactive electrodes for the simultaneous, non-enzymatic monitoring of sweat constituents.

## 2. Materials and Methods

### 2.1. Materials and Reagents

Indium tin oxide (ITO)-coated glass substrates (50 mm × 50 mm × 1.1 mm, 135 ± 15 nm thick ITO layer, ≤15 Ω sq^−1^) were procured from Saida Glass Co., Ltd. (Dongguan, China). Ferric chloride hexahydrate (FeCl_3_·6H_2_O, ACS, >97%), hydrochloric acid (HCl, ACS, 36.5–38.0%), titanium (IV) isopropoxide (TTIP, 98+%), and acetylacetone (AA, 99.0+%) were obtained from Thermo Scientific Chemicals (Waltham, MA, USA). Isopropyl alcohol (IPA, ≥99.8%, ChromAR, HPLC grade) and acetone (ACS, 99.5+) were supplied by Macron Fine Chemicals (Shanghai, China). Sodium chloride (NaCl, ≥99.0%), urea (ACS, 99.0–100.5%), D,L-lactic acid (LA, ≥85.0%), and potassium chloride (KCl, ≥99.0%) were purchased from Alfa Aesar (Karlsruhe, Germany).

### 2.2. Fabrication of Patterned TiO_2_|ITO Electrodes

**Figure 1:**
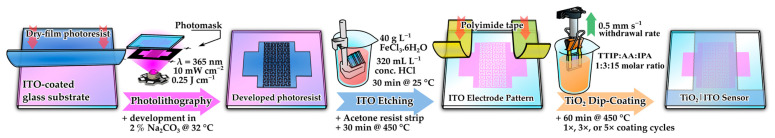
Technological steps employed to prepare the TiO_2_|ITO sensors used in this study.

The technological scheme employed for the preparation of TiO_2_-coated ITO sensor electrodes (TiO_2_|ITO) is presented in Figure 1. The fabrication process consisted of two main stages: photolithography and etching of the ITO-coated substrates, followed by dip-coating deposition of the TiO_2_ overlayer.

The ITO-coated glass substrates were cut into 25 × 25 mm squares. A negative dry-film photoresist (40 µm, MAH-115 type) was applied by roll lamination at 110 °C. The readout electrode patterns were transferred to the photoresist by contact photolithography using a UV illumination dose of 0.25 J cm^−2^ (UVA LED, λ = 365 nm, 10 mW cm^−2^), and the exposed photoresist was developed in a stream of warm 2 wt.% aqueous Na_2_CO_3_ solution (32 °C). The unprotected ITO areas were subsequently wet-etched in an aqueous etchant composed of 320 mL L^−1^ HCl and 40 g L^−1^ FeCl_3_·6H_2_O for 30 min at 25 °C. The remaining photoresist was removed with acetone, after which the patterned ITO substrates were annealed at 450 °C for 30 min to remove residual organics prior to TiO_2_ coating.

The TiO_2_ overlayer was deposited by sol–gel dip-coating. The sol was prepared from titanium (IV) tetraisopropoxide (TTIP), acetylacetone (AA), and isopropyl alcohol (IPA) at a TTIP:AA:IPA molar ratio of 1:3:15. Further details regarding the preparation and characterization of this formulation are provided in a previous publication [62]. The sol was deposited onto the patterned ITO substrates by dip-coating in a home-built apparatus at a withdrawal rate of 0.5 mm s^−1^. Prior to dip-coating, the contact pads of the two electrodes were protected with polyimide adhesive tape to prevent TiO_2_ deposition (see Figure 1). After each coating cycle, the polyimide tape was removed, and the samples were annealed at 450 °C for 60 min (heating rate 2 °C min^−1^) to convert the sol–gel layer into TiO_2_. One, three, or five consecutive coating cycles were applied to obtain TiO_2_|ITO sensors with increasing TiO_2_ thickness, denoted throughout the manuscript as 1×TiO_2_|ITO, 3×TiO_2_|ITO, and 5×TiO_2_|ITO, respectively. A total of 24 devices were fabricated in batches of four for each electrode topology. Two batches were used for electrochemical measurements, while one batch was reserved for structural characterization.

#### Geometry of the Readout Electrode Topologies

**Figure 2:**
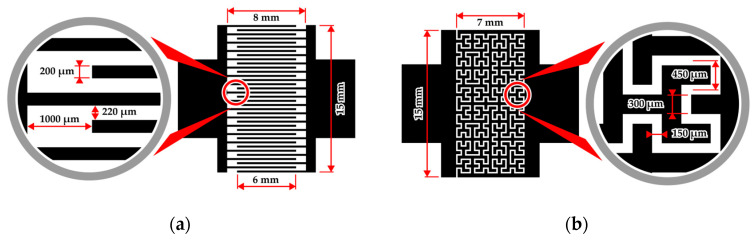
Schematic representation of the two-electrode topologies employed to form the readout electrodes in the TiO_2_|ITO sensors: (**a**) interdigitated electrode (IDE) topology; (**b**) Hilbert fractal electrode (HFE) topology.

The geometries of the two-electrode topologies studied in this work are shown in Figure 2. Throughout the manuscript, these configurations are referred to as the interdigitated electrode (IDE) and the Hilbert fractal electrode (HFE).

In both cases, the electrode pattern was embedded within a 15 × 10 mm rectangular area and featured two symmetric 8 × 4 mm contact pads protruding from each side (see Figure 2).

The IDE topology (Figure 2a) was formed by interdigitating two comb structures, each consisting of 18 fingers, with a busbar-to-busbar distance of 8 mm and a finger trace width of 200 µm. The finger length was 7 mm, resulting in a 6 mm central overlapping region between opposing fingers with a gap spacing of 220 µm and a finger-to-opposite-busbar distance of 1000 µm. The length of the interelectrode gap ($L_{gap}^{IDE}$) can be estimated by summing the overlapping segments between adjacent fingers belonging to opposite comb electrodes. For an IDE consisting of N finger pairs, this yields a total of (2N−1) parallel gap segments of length l, giving the following:

(1) $$L_{gap}^{IDE}=\left(2N-1\right)l,$$

where N is the number of finger pairs and l is the overlap length, yielding $L_{gap}^{IDE}$ = 210 mm. For simplicity of interpretation, the contribution of the finger termini to $L_{gap}^{IDE}$ was not included due to the larger gap spacing (1000 µm) in this region. However, its contribution, estimated from the number of fingers and the trace width, is approximately 7.2 mm (~3.5%) and can therefore be considered negligible. The conductive coated area $A_{ITO}^{IDE}$ was estimated from the number of fingers and the overall finger length, with the trace width to be 80.4 mm^2^ (including the two 15 mm^2^ busbars).

The HFE topology (Figure 2b) was constructed as a 2 × 4 array of eight third-order Hilbert units, arranged through rotational symmetry to form a continuous interelectrode gap of 150 µm. For a Hilbert curve of iteration n, the total curve length increases according to a recursive scaling law proportional to $4^{n}$ [63,64]. An order-n approximating polygon consists of $4^{n}$ nodes connected by $4^{n}-1$ linear segments. For a lattice spacing s (defined as the center-to-center spacing of the first-order U-cell; see Figure 2b), the total centerline length of a single tile is therefore $\left(4^{n}-1\right)s$. Thus, the total gap length ($L_{gap}^{HFE}$) of the eight-cell HFE can be estimated as

(2) $$L_{gap}^{HFE}=8\left(4^{n}-1\right)s,$$

where n = 3 and s = 450 µm for the present topology. This yields $L_{gap}^{HFE}$ ≈ 227 mm. The conductive coated area ($A_{ITO}^{HFE}$) was estimated by multiplying $L_{gap}^{HFE}$ by the gap width (150 µm) and subtracting this value from the geometric busbar-to-busbar area (15 × 10 mm). This calculation yields $A_{ITO}^{HFE}$ = 115.98 mm^2^, including the busbars.

All of the abovementioned parameters, derived from the photomask design, together with other relevant geometrical characteristics of the two-electrode topologies are summarized in Table 1. These parameters determine the effective electrolyte current path length and the wetted conductive area, both of which directly influence the measured impedance response of the sensors.

### 2.3. Electrochemical Impedance Spectroscopy Measurements

**Figure 3:**
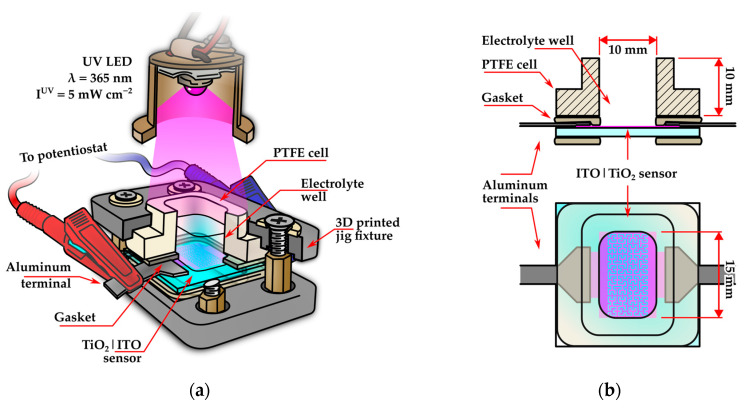
Schematic of the electrochemical cell used for electrochemical impedance spectroscopy measurements of the TiO_2_|ITO electrodes in synthetic sweat: (**a**) cutaway view showing the internal structure and jig fixture; (**b**) cross-sectional view of the electrolyte well and top view of the gasket and wetted electrode surface.

Electrochemical impedance spectroscopy (EIS) was used to investigate the response of the TiO_2_|ITO sensors in artificial sweat electrolytes. All experiments were conducted in a custom-built electrochemical cell, schematically illustrated in Figure 3. The cell consists of a PTFE sleeve serving as an electrolyte well, which is pressed onto a gasket positioned on top of the TiO_2_|ITO sensor surface using a 3D-printed jig fixture (Figure 3a). The cell is open-topped, allowing rapid replacement of the tested electrolyte using an automatic micropipette and enabling UV-assisted photoelectrochemical measurements by illumination with a UV LED (LTPL-C034UVH365, λ = 365 ± 5 nm, LiteOn Optoelectronics, Taipei, Taiwan). The LED provided an illumination intensity of 5 ± 0.15 mW cm^−2^ at the sample surface, measured through the electrolyte layer using a calibrated thermopile power meter (Thorlabs PM400 with S175C thermopile detector, Thorlabs, Newton, NJ, USA). Electrical contact to the contact pads of the TiO_2_|ITO sensors was established using 50 µm thick Al foil interconnects pressed through the gasket. The electrolyte well had internal dimensions of 15 × 10 mm, matching the active electrode area. However, due to the tooling used in PTFE routing, an inner filet with a 3 mm radius was present at the corners, occluding approximately 7.7 mm^2^ of the busbar area (see Figure 3b).

Artificial sweat electrolytes were prepared using an EN 1811:2011-type formulation [65], with a base composition of 40 mM NaCl, 17 mM urea, and 11 mM DL-lactic acid (LA). The formulation was modified by the addition of 5 mM KCl to provide a constant ionic background when Na^+^ concentration is varied and to reflect the typical millimolar potassium levels present in human eccrine sweat [4,12]. Three series of sweat compositions were prepared: Na^+^ varied in the range 0–100 mM, LA in the range 2.5–30 mM, and urea in the range 5–50 mM. All compositions were prepared from stock solutions of 2 M NaCl, 1 M urea, 1 M LA, and 1 M KCl, which were mixed in the desired ratios and adjusted to approximately 90% of the final volume. The pH was then adjusted to 6.5 ± 0.5 using 0.5 M NH_3_ prior to dilution to the final volume. The conductivity of all formulations was measured using a calibrated conductometer (model HI 98304, Hanna Instruments, Woonsocket, RI, USA). The compositions and measured conductivities are listed in Table S1 in the Supplementary Materials.

EIS measurements were performed using an EmStat Pico potentiostat module (PalmSens BV, Houten, The Netherlands) operated in a two-electrode configuration. All measurements were conducted at open-circuit potential (OCP) using a sinusoidal alternating current (AC) perturbation of 10 mV. The frequency range spanned 10^5^–1 Hz with 10 points per decade. Measurements were performed on at least two replications or on two independent devices. Error bars in all figures represent the standard deviation.

### 2.4. Characterization Methods and Data Analysis Tools and Software

Optical microscopy images were obtained using an IM-3MET metallographic microscope equipped with a 10 Mpix CMOS camera (C-B10+, Optika S.r.l., Ponteranica, Bergamo, Italy). Scanning electron microscopy (SEM) micrographs were acquired using a TM4000 microscope (Hitachi, Tokyo, Japan) operating at 15 kV with a back-scattered electron (BSE) detector. Atomic force microscopy (AFM) measurements were performed on a Dimension 3100 system with a Nanoscope IIIa controller (Veeco Instruments Inc., Plainview, NY, USA), operating in tapping mode using a Tap300Al-G probe (BudgetSensors, Innovative Solutions Bulgaria Ltd., Sofia, Bulgaria). Surface morphology was characterized using 1 × 1 μm scans acquired at a scan rate of 0.9 Hz, while 30 × 30 μm scans were obtained at 0.3 Hz for step height measurements.

UV–Vis transmittance spectra were recorded using an SP-V1100 spectrophotometer (DLAB Scientific Co., Ltd., Beijing, China). X-ray diffraction (XRD) patterns were obtained using a Bruker D8 Advance diffractometer (Bruker AXS GmbH, Karlsruhe, Germany) equipped with a Cu Kα radiation source and a LynxEye detector. Raman spectra were recorded using a TO-ERS-532 spectrometer (Thunder Optics S.A.S., Montpellier, France) equipped with a 20× microscope objective Raman probe and a 532 nm excitation source.

Image processing and dimensional measurements were carried out using Fiji v.2.16.0/1.54p [66]. AFM data were processed using Gwyddion v.2.68 [67]. Raman spectra were processed using Spectragryph v.1.2.16.1. General data processing and figure preparation were performed using R version 4.5.2 (31 October 2025) [68].

Electrochemical data acquisition and preliminary equivalent circuit fitting were performed using PSTrace 5 (v.5.9.2317). The obtained parameter estimates were then used as starting values for automated EIS fitting using custom R scripts employing the minpack.lm package for multivariate nonlinear least-squares fitting [69]. Lithography mask designs and publication artwork were created using Inkscape v.1.4.2.

## 3. Results and Discussion

### 3.1. Characterization of the TiO_2_|ITO Sensors

**Figure 4:**
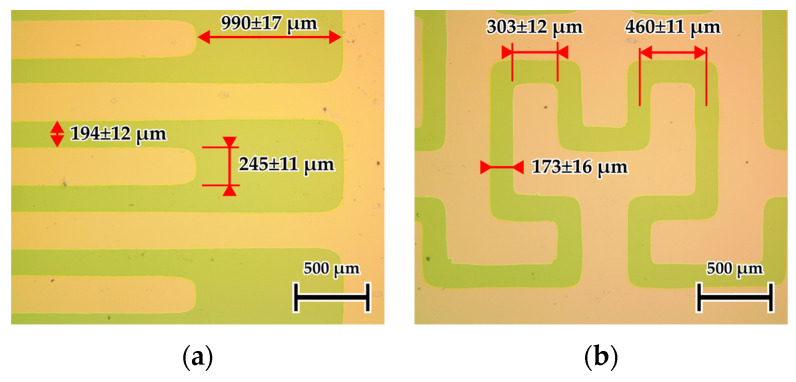
Optical microscopy images of the etched ITO patterns: (**a**) interdigitated electrode (IDE) topology; (**b**) Hilbert fractal electrode (HFE) topology.

Optical microscopy was used to verify the transfer of the photolithographic mask design onto the etched ITO surface. Representative micrographs of the two-electrode topologies are shown in Figure 4. In both cases, the overall pattern was well reproduced, although slight edge rounding of the square features was observed.

A comparison between the nominal photomask dimensions and the etched structures revealed deviations primarily affecting the fine lateral features. For the IDE topology (Figure 4a), the conductive traces were broadened to 244.9 ± 11.3 µm compared to the nominal 200 µm, while the interelectrode gap decreased to 193.8 ± 11.7 µm (nominal 220 µm). In contrast, the larger terminal spacing remained close to the design value (989.9 ± 17.3 µm, nominal 1000 µm).

For the fractal topology (Figure 4b), the trace width (302.8 ± 11.7 µm) and cell size (459.8 ± 10.9 µm) closely matched the photomask design, while the narrow gaps were slightly enlarged (172.6 ± 15.5 µm, nominal 150 µm). These deviations are attributed to the combined effects of mask fabrication, contact lithography transfer, and isotropic edge rounding during wet etching. The experimentally measured geometrical parameters were therefore used in the numerical simulations presented later.

**Figure 5:**
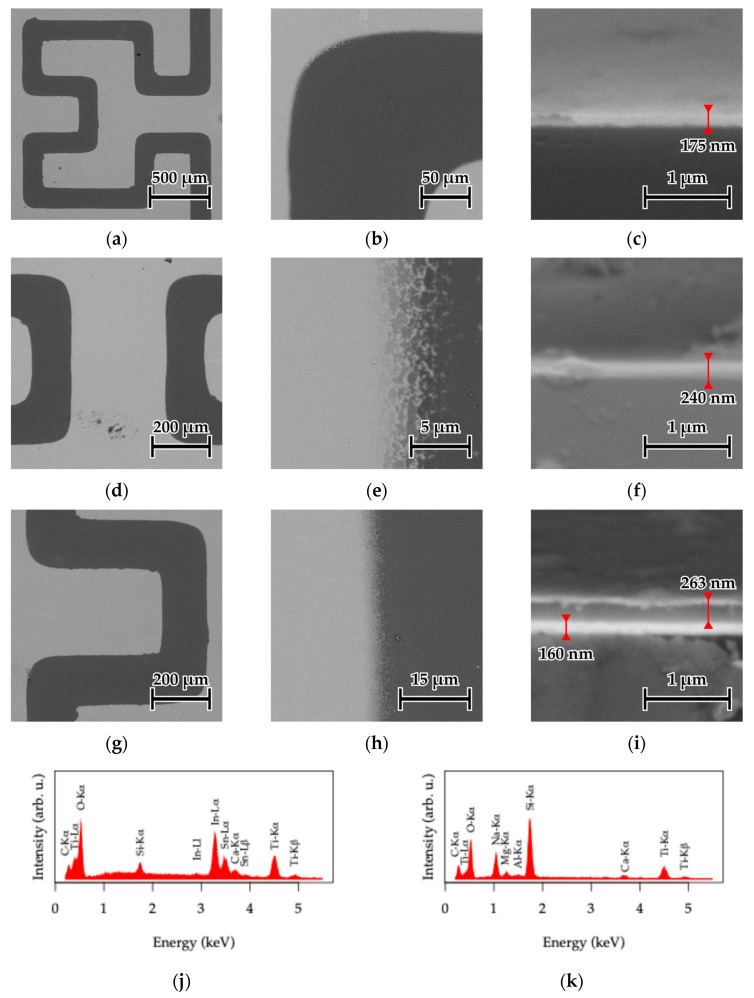
SEM images showing top view morphology at different magnifications and cross-sections of the HFE ITO structures for the (**a**–**c**) 1×TiO_2_|ITO sensor, (**d**–**f**) 3×TiO_2_|ITO sensor, and (**g**–**i**) 5×TiO_2_|ITO sensor. EDS spectra obtained from point measurements on (**j**) unetched ITO regions, and (**k**) etched areas for the 5×TiO_2_|ITO sample.

SEM analysis was performed to examine the morphology of the dip-coated 1×, 3×, and 5×TiO_2_|ITO electrodes. Representative micrographs are shown in Figure 5. Low-magnification images (Figure 5a,d,g) demonstrate that the dip-coating process produced continuous TiO_2_ layers without visible delamination, cracks or large defects, even for the thickest coating.

Higher-magnification images near the etched ITO boundary (Figure 5b,e) show well-defined pattern edges with minor undulations originating from the etching process, indicating overall good pattern fidelity.

Overall, the SEM observations confirm that the TiO_2_ layers are uniform and conformally cover the patterned ITO surface.

Cross-sectional SEM images (Figure 5c–i) reveal a clearly distinguishable ITO layer with a thickness of approximately 175 nm. The TiO_2_ overlayer is difficult to resolve in the 1×TiO_2_|ITO and 3×TiO_2_|ITO samples (Figure 5c,f), although a slight increase in the total layer thickness is observed in the latter case (~240 nm). In contrast, the TiO_2_ overlayer becomes clearly visible in the 5×TiO_2_|ITO sample (Figure 5i), where a distinct ITO/TiO_2_ interface can be identified using BSE contrast. The TiO_2_ thickness in this case is approximately 263 nm, corresponding to an average deposition rate of ~50 nm per coating cycle, consistent with previous reports for the same sol–gel system [62]. To confirm complete etching of the ITO, EDS point measurements were performed on conductive regions (bright areas in Figure 5) and etched regions (dark areas). The spectra (Figure 5j,k) show prominent In-Lα and Sn-Lα peaks in the former, which are absent in the latter, while the Ti-Kα signal from TiO_2_ is present in both cases.

**Figure 6:**
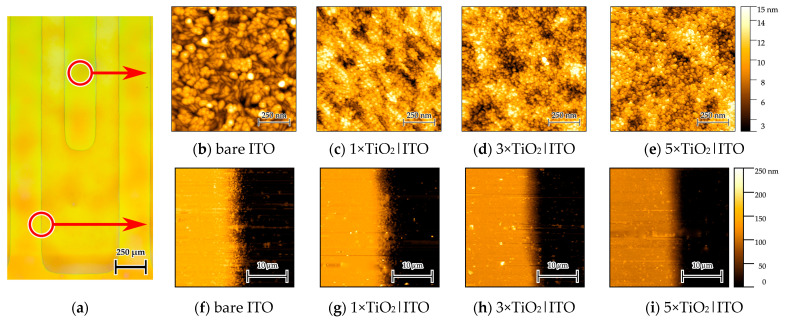
AFM images of the TiO_2_|ITO sensors: (**a**) microscopy image indicating the measurement location; (**b**–**e**) surface topography of bare ITO and TiO_2_-coated samples; (**f**–**i**) corresponding step height profiles across the etched ITO edge.

To further examine the nanoscale surface morphology, AFM analysis was performed on the TiO_2_ layers. Surface topography and step height measurements across the etched ITO edge for the bare ITO and the 1×–5×TiO_2_|ITO samples are presented in Figure 6.

Figure 6a shows an optical microscopy image of the 3×TiO_2_|ITO (IDE) sample indicating the AFM measurement location. Figure 6b–e present AFM topography images acquired from the TiO_2_ overlayer deposited on the ITO traces, while Figure 6f–i show the corresponding step height profiles. AFM images of TiO_2_ deposited on the etched glass regions are provided in Figure S1 in the Supplementary Materials.

The bare ITO surface exhibits a morphology consisting of spherical features approximately 50 nm in diameter, characteristic of thermally evaporated ITO, with a root-mean-square (RMS) roughness of 4.8 nm (Figure 6b). After TiO_2_ deposition, a finer granular morphology appears, consisting of densely packed particles with characteristic sizes of ~20 nm. In the 1×TiO_2_|ITO sample (Figure 6c), this morphology partially follows the underlying ITO structure, while the surface roughness decreases to 2.3 nm. With increasing coating cycles (Figure 6d,e), the TiO_2_ layer progressively smooths the surface features, resulting in an RMS roughness of approximately 1.9 nm. The decrease in surface roughness upon TiO_2_ deposition is attributed to the conformal nature of the sol–gel coating, which progressively smooths the underlying ITO morphology rather than forming a particulate overlayer.

The step height measurements (Figure 6f–i) reveal a smooth transition region of approximately 5 µm width at the etched ITO edge, which remains preserved after TiO_2_ deposition. The step height decreases from 152 nm for the bare ITO to 138–108 nm for the 1×–5×TiO_2_|ITO series, reflecting progressive edge smoothing by the conformal TiO_2_ layer. Nevertheless, the step remains clearly distinguishable in all cases.

The extracted step height profiles are shown in Figure S2, and the numerical AFM parameters are summarized in Table S2 in the Supplementary Materials.

**Figure 7:**
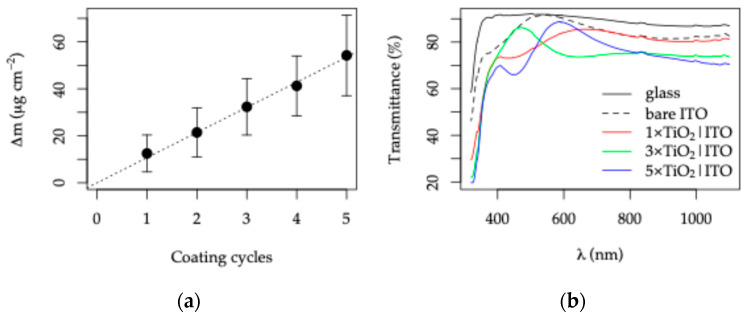
Characterization of the TiO_2_|ITO layers: (**a**) mass increase per TiO_2_ coating cycle; (**b**) UV–Vis transmittance spectra.

The TiO_2_ deposition rate was additionally determined gravimetrically by measuring the mass increase in the substrates after each dip-coating cycle and normalizing it to the coated geometric area. As shown in Figure 7a, the mass increased linearly with the number of coating cycles, corresponding to an average deposition rate of 10.7 µg cm^−2^ per cycle, consistent with previous reports for the same sol–gel system [62].

UV–Vis transmittance spectra (Figure 7b) show that all samples remain optically transparent, with transmittance values exceeding 60% across the visible range. The presence of well-defined interference fringes indicates the formation of continuous and optically uniform TiO_2_ films with low scattering losses, suggesting limited surface defects or cracking. The slight shift and damping of the fringes with increasing TiO_2_ thickness further confirm the progressive growth of the coating and the increase in optical path length.

**Figure 8:**
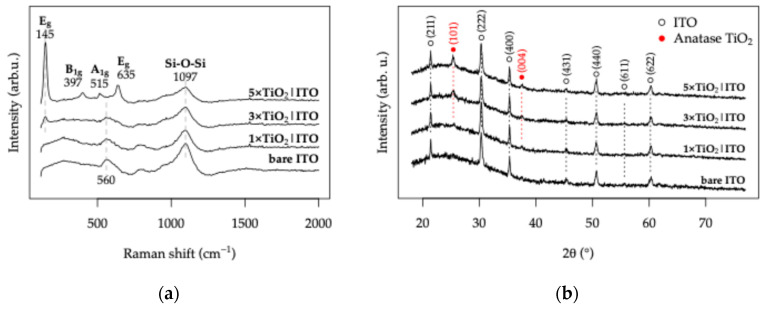
Phase identification of the TiO_2_ layer in TiO_2_|ITO electrodes: (**a**) Raman spectra; (**b**) XRD patterns.

The phase composition of the deposited TiO_2_ layers was examined using Raman spectroscopy and X-ray diffraction (Figure 8).

The Raman spectrum of the bare ITO-coated glass substrate exhibits a broad asymmetric band extending from approximately 520–660 cm^−1^, with a maximum at ~560 cm^−1^, which is commonly attributed to substrate-related scattering from the glass/ITO system [70]. A band at ~1097 cm^−1^ is also observed and assigned to Si-O-Si vibrations of the glass substrate [71].

With increasing TiO_2_ overlayer thickness (1×–5× dip-coating cycles), the characteristic Raman modes of TiO_2_ progressively emerge and increase in intensity, most prominently the E_g_ mode at ~144–145 cm^−1^, indicating the formation of anatase TiO_2_ [72]. For the thickest TiO_2_ film, additional anatase modes appear at ~397 cm^−1^ (B_1g_), ~515 cm^−1^ (A_1g_), and ~637 cm^−1^ (E_g_). At the same time, the substrate-related bands decrease in intensity due to attenuation by the increasingly Raman-dominant TiO_2_ overlayer (Figure 8a).

The formation of polycrystalline anatase TiO_2_ is further confirmed by XRD analysis (Figure 8b). The ITO layer is identified by reflections at 21.4°, 30.4°, 35.3°, 50.7°, and 60.2° (2θ), corresponding to the (211), (222), (400), (440), and (622) planes (PDF 01-089-4597). Scherrer analysis of the most intense ITO (222) peak yields an average crystallite size of 21.8 ± 0.9 nm, with no systematic variation across the sample series, indicating that repeated heat treatments at 450 °C do not induce recrystallization of the ITO layer.

The presence of anatase TiO_2_ is confirmed by the appearance of the (101) and (004) reflections at 25.4° and 37.5° (2θ) (PDF 03-065-5714). The crystallite size estimated from the TiO_2_ (101) peak is 14.2 ± 0.4 nm.

Overall, the characterization results demonstrate that dip-coating produces uniform, conformal TiO_2_ layers on the patterned ITO electrodes. The TiO_2_ overlayer exhibits polycrystalline anatase structure, smooth nanoscale morphology, and a thickness that increases monotonically with the number of coating cycles at approximately 50 nm per cycle.

### 3.2. Response and Electric Field Distribution of the TiO_2_|ITO Sensors in Distilled Water

To investigate the influence of the ITO electrode topology and the TiO_2_ layer thickness on the geometric capacitance of the sensors, electrochemical impedance spectroscopy (EIS) measurements were carried out in distilled water. Under these conditions, charge-transfer processes are suppressed, and the electrode stack behaves as a blocked electrode system.

**Figure 9:**
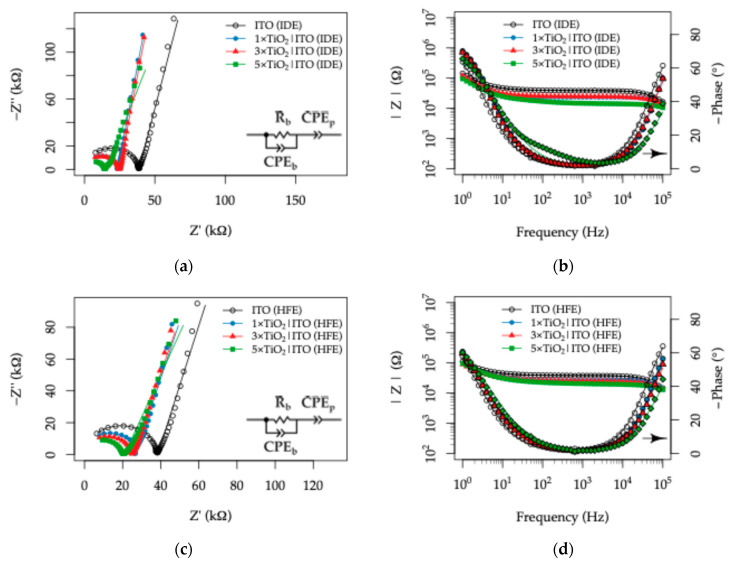
EIS measurements for the TiO_2_|ITO electrodes in distilled water without UV illumination: (**a**,**b**) Nyquist and corresponding Bode plots for the TiO_2_|ITO (IDE) topology; (**c**,**d**) Nyquist and corresponding Bode plots for the TiO_2_|ITO (HFE) topology.

The EIS results obtained for the IDE and HFE topologies with varying TiO_2_ layer thickness in darkness are presented in Figure 9, together with the equivalent circuit used to model the data. The circuit, shown as an inset, consists of a high-frequency branch composed of the bulk electrolyte resistance ($R_{b}$) and a constant phase element (${CPE}_{b}$) associated with the geometric capacitance of the electrode structure. This branch is connected in series with a low-frequency constant phase element (${CPE}_{p}$), representing interfacial double-layer polarization at the electrode surface [73].

All fitted values obtained from the equivalent circuit analysis are summarized in Table S3 in the Supplementary Materials.

For the bare ITO electrodes, the bulk resistance $R_{b}$, associated with the electrolyte resistance between the electrodes, is approximately 38 kΩ, and remains nearly identical for both topologies. However, upon deposition of TiO_2_, the $R_{b}$ value decreases to approximately 15 kΩ for the TiO_2_|ITO IDE sensors and 21 kΩ for the TiO_2_|ITO HFE sensors as the TiO_2_ thickness increases from 1× to 5× coating cycles. This trend suggests a modification of the conduction path within the electrode gap. A possible explanation is the formation of surface conductivity governed by the dissociation of adsorbed water molecules into hydroxyl ions, a mechanism commonly reported for TiO_2_-based humidity sensors [74]. Since distilled water effectively represents 100% humidity conditions, the contribution of adsorbed water at the TiO_2_ surface cannot be neglected. Notably, the decrease in $R_{b}$ is more pronounced for the IDE topology, even though this geometry exhibits a slightly larger interelectrode gap both in the photomask design and in the fabricated electrodes.

The geometric capacitance element ${CPE}_{b}$ remains relatively constant at approximately 10^−10^ F, although a gradual increase is observed with increasing TiO_2_ thickness, reaching approximately 9 × 10^−10^ F for the IDE and 5 × 10^−10^ F for the HFE topology. This increase may be associated with partial dielectric penetration of the electric field into the TiO_2_ layer. At the same time, the corresponding α parameter decreases from 0.96 to 0.83, indicating a deviation from ideal capacitive behavior. Since the surface roughness of the TiO_2_|ITO electrodes is lower than that of bare ITO, this effect is most likely related to increasing porosity within the thicker TiO_2_ layers.

The interfacial polarization element ${CPE}_{p}$ exhibits a more pronounced difference between the two-electrode topologies. While ${CPE}_{p}$ increases slightly with TiO_2_ thickness for both structures, the HFE topology consistently shows approximately 40% higher ${CPE}_{p}$ values compared to the IDE. Typical values of 2.6 × 10^−6^ F for HFE and 1.9 × 10^−6^ F for IDE were obtained for the TiO_2_|ITO sensors. This observation suggests that the fractal electrode geometry produces stronger interfacial polarization, likely due to a more heterogeneous electric field distribution.

UV illumination produces only a minor influence on the overall impedance response. The corresponding EIS spectra for the TiO_2_|ITO (IDE) and TiO_2_|ITO (HFE) sensors under UV illumination are presented in Figure S3 in the Supplementary Materials.

To further investigate the difference in electric field distribution between the two-electrode geometries, numerical simulations were performed using ANSYS-Maxwell 3D (2025 R1).

Three-dimensional electric transient analysis with Electric Scalar Potential (ESP) formulation is used for sensor electric field calculation. The governing time-dependent equation, according to ESP-*V*, in general form is

(3) $$\nabla \left(\epsilon \nabla \frac{\partial V}{\partial t}\right)+\nabla \left(\sigma \nabla V\right)=0,$$

where *ε* and *σ* are electric permittivity and electric conductivity of materials, respectively.

This formulation takes into account both electric permittivity and the conductivity of materials in time-dependent electric field distribution calculation.

Electric field intensity **E** can be calculated as a gradient of the electric scalar potential,

(4) E=−∇V.

**Figure 10:**
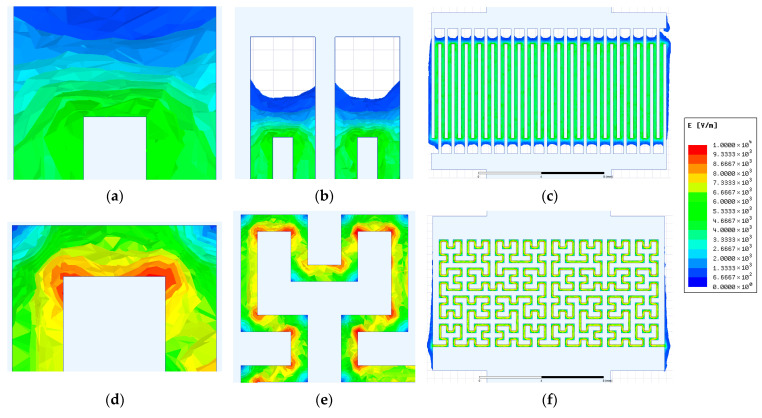
Electric field distribution for the ITO readout electrodes based on the photomask design: (**a**–**c**) interdigitated electrodes (IDEs); (**d**–**f**) Hilbert fractal electrodes (HFEs).

Field results are presented in dielectric domains between electrodes as electric field intensity distribution plots. The resulting field distributions for the IDE and HFE patterns based on the original photomask design are presented in Figure 10.

Several observations can be made from the numerical simulations. First, for the IDE topology, the electric field intensity decreases sharply in the extended termini regions, where the interelectrode gap reaches 1000 µm (Figure 10a). In these regions, the field penetration extends only to approximately half of the nominal gap distance, indicating that they contribute only marginally to the overall sensor response. This observation supports the simplification adopted in Section Geometry of the Readout Electrode Topologies, where these regions were excluded from the gap length estimation.

Second, the HFE topology exhibits significantly stronger local electric field concentrations near the electrode edges, with field densities approaching nearly an order of magnitude higher than those observed for the IDE configuration (Figure 10e compared to Figure 10b). The non-uniformity of the electric field distribution becomes even more apparent when the sensor-level simulations are considered (Figure 10f versus Figure 10c), confirming that this behavior is present across the entire electrode array.

**Figure 11:**
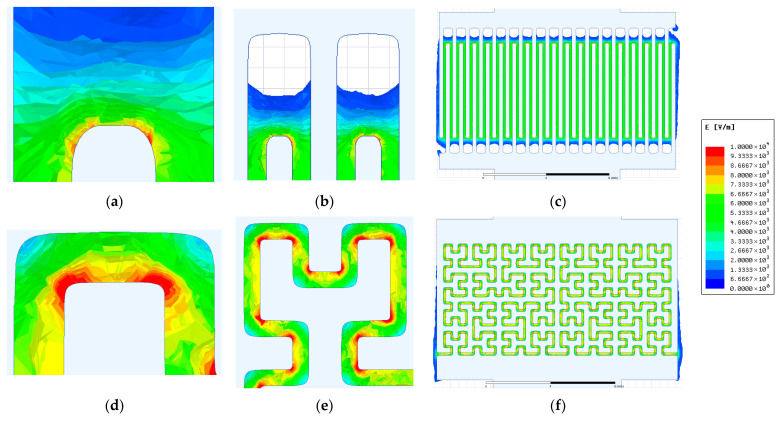
Electric field distribution for the ITO readout electrodes based on geometries reconstructed from optical microscopy measurements: (**a**–**c**) IDE topology; (**d**–**f**) HFE topology.

It should be noted, however, that the fabricated electrodes exhibit rounded edges and slightly modified dimensions, as shown in the optical microscopy images in Figure 4, compared to the ideal photomask design. To account for this effect, the simulated electrode geometries were modified according to the experimentally measured dimensions and edge radii obtained from optical microscopy. The resulting electric field distributions are presented in Figure 11.

The simulations show that the overall electric field topology remains largely unchanged, with nearly identical current pathways between the electrodes. Comparison of the local field distributions (Figure 11a,b,d,e versus Figure 10a,b,d,e) reveals some differences in the electric field concentration near the electrode edges, particularly for the IDE topology. However, when considering the sensor-level distributions (Figure 11c,f versus Figure 10c,f), the macroscopic current paths remain essentially the same.

Therefore, although the observed edge rounding in the fabricated electrodes modifies the local field concentration, the overall impedance behavior of the sensors can be attributed primarily to the electrode layout rather than to the specific corner geometry.

### 3.3. Impedimetric Response of the TiO_2_|ITO Sensors in Synthetic Sweat

#### 3.3.1. Effect of UV Illumination

**Figure 12:**
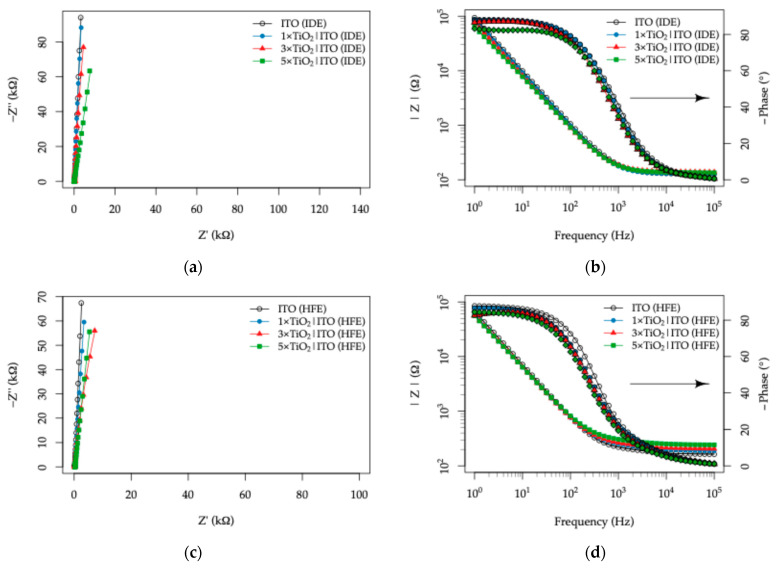
Electrochemical impedance spectroscopy (EIS) response of the TiO_2_|ITO sensors with varying TiO_2_ thickness measured in artificial sweat (40 mM Na^+^, 11 mM lactic acid, 17 mM urea) under dark conditions: (**a**) Nyquist plot and (**b**) corresponding Bode plot for the interdigitated electrode (IDE) topology; (**c**) Nyquist plot and (**d**) corresponding Bode plot for the Hilbert fractal electrode (HFE) topology.

The response of the as-fabricated TiO_2_|ITO sensors to the composition of the synthetic sweat solutions was evaluated by electrochemical impedance spectroscopy (EIS). As a starting point, the behavior of the two-electrode topologies, namely the IDE and HFE patterns, was examined in the standard EN 1811 artificial sweat composition [65] containing 40 mM Na^+^, 11 mM lactic acid (LA), and 17 mM urea, under dark conditions. The corresponding Nyquist and Bode plots for the IDE and HFE topologies are presented in Figure 12a,b and Figure 12c,d, respectively.

**Figure 13:**
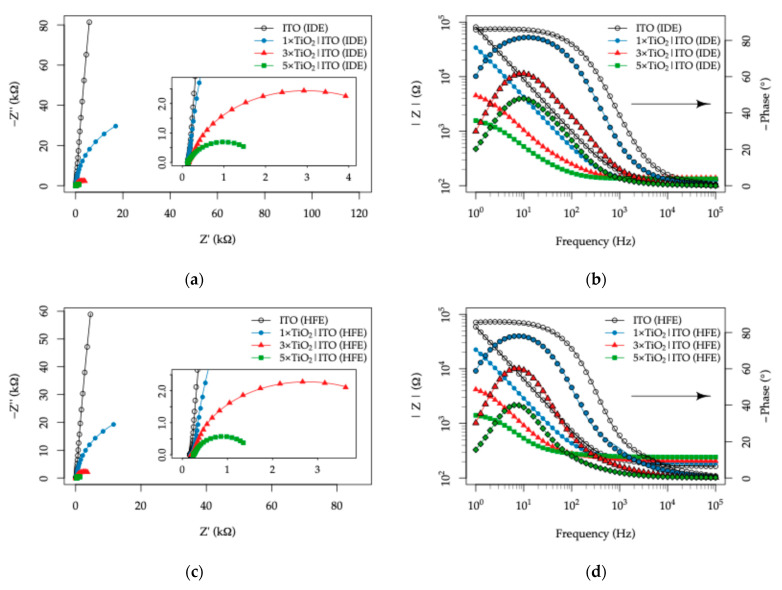
Electrochemical impedance spectroscopy (EIS) response of the TiO_2_|ITO sensors with varying TiO_2_ thickness measured in artificial sweat (40 mM Na^+^, 11 mM lactic acid, 17 mM urea) under UV illumination: (**a**) Nyquist plot and (**b**) corresponding Bode plot for the interdigitated electrode (IDE) topology; (**c**) Nyquist plot and (**d**) corresponding Bode plot for the Hilbert fractal electrode (HFE) topology.

In darkness, all TiO_2_|ITO electrodes exhibited similar impedance responses over the 10^5^–1 Hz frequency range, with comparably open Nyquist plots. This behavior indicates negligible charge transfer at the electrode–electrolyte interface, together with only minor effects of electrode topology and TiO_2_ layer thickness on the overall response. Under UV illumination, however, a pronounced change in the EIS behavior was observed, revealing a strong dependence on both the ITO readout electrode topology and the TiO_2_ layer thickness, as shown in Figure 13.

Relative to the bare ITO electrodes, all TiO_2_-coated samples exhibited a monotonic decrease in semicircle radius in the Nyquist plots under UV illumination, with the magnitude of this effect increasing with TiO_2_ thickness. In the 1×TiO_2_|ITO electrodes, the semicircle remained only weakly developed, whereas in the 3×TiO_2_|ITO and 5×TiO_2_|ITO electrodes, an almost complete semicircle was observed for both topologies. Concomitantly, the Bode plots showed attenuation of both the impedance modulus and the phase maximum. When the two topologies are compared, these UV-induced effects are more pronounced for the HFE topology than for the IDE topology.

**Figure 14:**
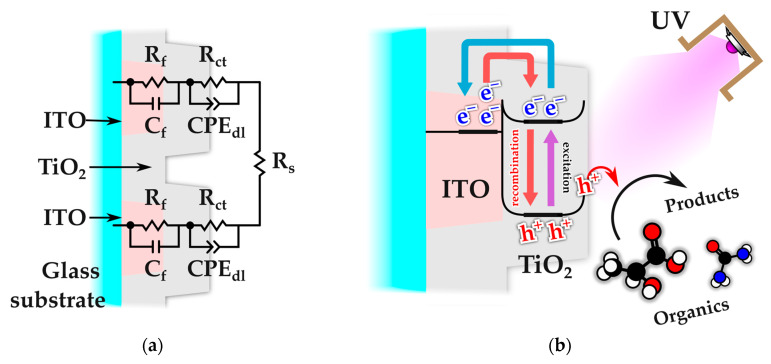
Schematic representation of the TiO_2_|ITO electrode: (**a**) equivalent circuit used for fitting the EIS response of the symmetric cell in artificial sweat, consisting of a high-frequency bulk $(R_{f}|\left|C_{f}\right)$ and a low-frequency interfacial $(R_{ct}|\left|{CPE}_{dl}\right)$ branches in series with the solution resistance $R_{s}$; (**b**) illustration of the photoinduced processes at the TiO_2_|ITO interface, including electron transfer, recombination, and interaction with electrolyte species.

To obtain quantitative information on the effects of topology and TiO_2_ thickness, the impedance data were analyzed by equivalent circuit modeling. In contrast to distilled water, the artificial sweat electrolyte possesses both significant ionic conductivity and an appreciable content of organic components, namely lactic acid and urea. Under UV illumination, these characteristics may enable interfacial charge-transfer processes through interaction with photogenerated charge carriers in the TiO_2_ layer. Since the electrochemical cell consists of two symmetric TiO_2_|ITO electrodes, both in contact with the electrolyte through the TiO_2_ overlayer, as illustrated in Figure 14a, each electrode can be represented by a two-branch RC circuit connected in series with the solution resistance, $R_{s}$.

More specifically, each electrode is described by a high-frequency branch, consisting of $R_{f}$ in parallel with $C_{f}$, which accounts for the bulk contribution of the TiO_2_|ITO film stack, and a low-frequency branch, consisting of $R_{ct}$ in parallel with a constant phase element, ${CPE}_{dl}$, which represents the charge-transfer resistance and the non-ideal double-layer capacitance at the film stack–electrolyte interface. Owing to the symmetric cell configuration, and in order to reduce the number of adjustable fitting parameters, the equivalent circuit was implemented as

(5) $$Z_{cell}=R_{s}+2[(R_{f}|\left|C_{f}\right)+(R_{ct}|\left|CPE_{dl}\right)]$$

Owing to the symmetric configuration of the two-electrode cell, the impedance response is modeled as two identical electrode–electrolyte interfaces connected in series, resulting in the factor of two in Equation (5). In this representation, the fitted parameters ($R_{ct}$ and ${CPE}_{dl}$) correspond to a single TiO_2_–electrolyte interface, while the total measured impedance reflects the combined contribution of both electrodes. This approach allows the interfacial properties of one electrode to be evaluated, assuming identical behavior at both interfaces.

The electrochemical response under UV illumination originates from the photogeneration of electron–hole pairs in TiO_2_ upon excitation of valence-band electrons to the conduction band [55,56]. About 99% of the photogenerated charge carriers recombine [75], while the rest participate in interfacial redox reactions with species present in the electrolyte, including the oxidation of water or adsorbed organic molecules, such as lactic acid or urea [76,77], by photogenerated holes and the reduction in dissolved molecular oxygen by electrons [57]. When TiO_2_ is deposited onto ITO, the higher work function of the ITO substrate facilitates the transfer of photogenerated electrons from the TiO_2_ conduction band into the conductive layer [78], resulting in partial charge separation and an increased lifetime of photogenerated holes.

Under open-circuit conditions, the accumulated electrons in the ITO layer and holes in the TiO_2_ establish a steady-state population governed by the balance between recombination and interfacial charge-transfer processes. In this context, the charge-transfer resistance $R_{ct}$ reflects the effective resistance associated with these interfacial processes, which are largely controlled by recombination kinetics in the absence of strongly reactive species. Suppression of recombination through interactions with electrolyte components therefore leads to a decrease in $R_{ct}$, while enhanced charge accumulation at the interface contributes to an increase in the measured interfacial capacitance. These processes are schematically illustrated in Figure 14b.

Accordingly, all fitted parameters discussed in the following subsections are reported on a per-electrode basis. The effects of electrode topology and TiO_2_ thickness on the EIS response to the individual synthetic sweat composition parameters are discussed in the following sections.

#### 3.3.2. Influence of Na^+^ Concentration on the Impedimetric Response

The bare ITO and TiO_2_-coated electrodes (1×TiO_2_|ITO–5×TiO_2_|ITO) with IDE and HFE topologies were exposed to artificial sweat solutions containing 0–100 mM Na^+^. The complete set of raw EIS spectra together with the corresponding equivalent circuit fits (Figure 14) and extracted fitting parameters are provided in the Supplementary Materials. Specifically, the results for both topologies under dark conditions are presented in Figures S4 and S5 with the associated parameters summarized in Table S4, while the measurements under UV illumination are shown in Figures S6 and S7 with the corresponding parameters listed in Table S5.

**Figure 15:**
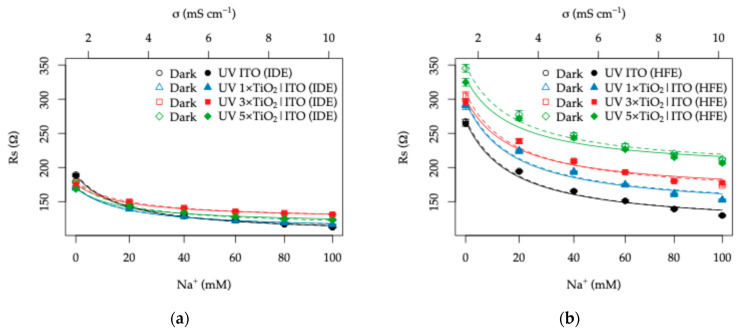
Dependence of the electrolyte resistance term ($R_{s}$) on Na^+^ concentration (0–100 mM) in artificial sweat (11 mM lactic acid, 17 mM urea, 5 mM background K^+^). A secondary *x*-axis indicates the corresponding electrolyte conductivity (σ) measured using a calibrated conductometer. Data are shown for (**a**) interdigitated electrode (IDE) patterned ITO and (**b**) Hilbert fractal electrode (HFE) patterned ITO.

Starting with the functional dependence of the $R_{s}$ parameter obtained from the equivalent circuit fits (Figure 14), its variation with Na^+^ concentration for both electrode topologies and all TiO_2_ layer thicknesses is shown in Figure 15a,b. A complete overlap between the dark and UV datasets is observed, confirming that UV illumination has a negligible influence on the high-frequency solution resistance. In both topologies, $R_{s}$ decreases monotonically with increasing Na^+^ concentration, reflecting the corresponding increase in electrolyte conductivity (σ) and indicating that the conductivity of artificial sweat can be reliably monitored regardless of the illumination condition.

Although $R_{s}(\sigma )$ dependence is nonlinear, this behavior is characteristic of planar conductometric cells with non-parallel electrode geometries [34,61]. According to classical interdigitated electrode theory, the bulk electrolyte resistance follows $R_{s}=K\rho$, where ρ=1/σ and K represents the cell constant [24,25]. Accordingly, the experimental data were fitted using the empirical relation:

(6) $$R_{s}\left(\sigma \right)=\frac{K}{\sigma }+R_{0},$$

where K represents a topology-dependent geometry factor and $R_{0}$ accounts for conductivity-independent parasitic contributions arising from the ITO layer and TiO_2_-induced current constriction effects.

The extracted parameters (Table 2) reveal a pronounced topology dependence. The HFE configuration exhibits approximately two-fold larger K values compared to the IDE topology, indicating a longer effective ionic current path within the electrolyte. This behavior is consistent with the electric field simulations presented in Section 3.2 and with previous observations for fractal electrode geometries, which increase perimeter density and redistribute electric field lines in the plane, leading to modified current trajectories [34,61]; it also reflects macroscopic redistribution of the electric field and ionic current pathways imposed by the electrode geometry, rather than nanoscale interfacial effects. In contrast, classical IDE geometries confine the electric field predominantly between adjacent opposing fingers, resulting in a comparatively shorter effective ionic path and therefore a smaller geometry factor [24,25].

For the bare ITO case, the ratio K(HFE)/K(IDE) is 1.76. Upon TiO_2_ overcoating, this ratio increases to approximately 2.5 and remains largely independent of thickness, indicating that the topology-driven redistribution of the electric field remains the dominant factor governing the electrolyte resistance. In terms of sensitivity, the $R_{s}$ change corresponds to ~0.24–0.40% mM^−1^ (IDE) and ~0.40–0.52% mM^−1^ (HFE) in the 0–100 mM Na^+^ range, confirming the stronger response of the fractal design.

The IDE configuration shows a moderate decrease in K with increasing TiO_2_ thickness (from 141 to 105 Ω mS cm^−1^ in dark conditions), accompanied by slightly lower values under UV illumination, suggesting a certain sensitivity to boundary modifications at the electrode surface. In contrast, the HFE topology exhibits no clear systematic dependence of K on TiO_2_ thickness, further supporting the notion that its field distribution is primarily governed by the lateral electrode geometry rather than by vertical boundary effects.

Meanwhile, the $R_{0}$ term increases with increasing TiO_2_ thickness, particularly for the HFE topology. This trend can be attributed to enhanced in-plane current spreading resistance within the patterned ITO layer together with additional current constriction effects introduced by the thicker TiO_2_ overlayer. The ratio $R_{0}(HFE)/R_{0}(IDE)$ increases progressively from 1.12 to 1.71 with increasing TiO_2_ thickness, consistent with amplified spreading resistance contributions in the fractal electrode configuration.

In addition to the solution resistance $R_{s}$, the equivalent circuit contains two parallel branches, ($R_{f}||C_{f}$) and $\left(R_{ct}||{CPE}_{dl}\right)$, which describe interfacial processes occurring at the electrode–electrolyte boundary. The fitted parameters associated with these branches are summarized in the Supplementary Materials (Tables S4 and S5). The parameters of the ($R_{f}||C_{f}$) branch remain relatively stable across the investigated conditions. The fitted $R_{f}$ values are typically on the order of several tens of ohms, significantly smaller than the electrolyte resistance $R_{s}$, indicating that this branch contributes mainly through its capacitive component $C_{f}$. The capacitance $C_{f}$, on the order 10^−6^–10^−5^ F, primarily reflects the geometric capacitance of the electrode structures and shows a systematic dependence on electrode topology, with larger values observed for the IDEs compared to the HFE structures. This behavior is consistent with the EIS results obtained in distilled water, where the absence of electroactive species resulted in a predominantly capacitive response governed by the geometric capacitance of the electrode configuration.

In contrast, the $\left(R_{ct}||{CPE}_{dl}\right)$ branch exhibits a markedly different behavior. Under dark conditions, the fitted $R_{ct}$ values are extremely large (≥10^6^ Ω), indicating a nearly blocking interface with only a minor contribution to the overall impedance response. Upon UV illumination, however, $R_{ct}$ decreases by several orders of magnitude due to the activation of photocatalytic processes at the TiO_2_ surface, and this branch becomes the dominant contributor to the impedance spectra. For this reason, the following discussion focuses primarily on the parameters $R_{ct}$ and ${CPE}_{dl}$, which reflect the photoinduced interfacial charge-transfer processes governing the sensor response.

**Figure 16:**
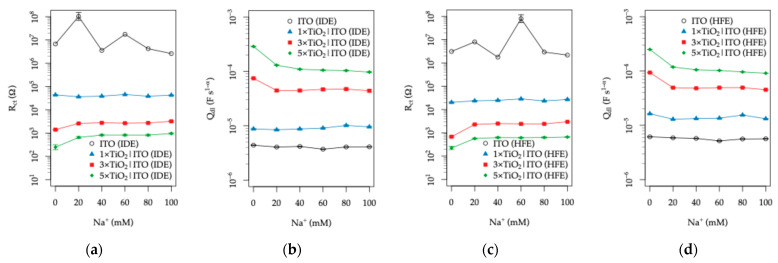
Dependence of the fitted interfacial charge-transfer parameters on Na^+^ concentration under UV illumination for artificial sweat solutions (11 mM lactic acid, 17 mM urea, 5 mM background K^+^): (**a**) charge-transfer resistance $R_{ct}$ for the IDE topology; (**b**) derived double-layer capacitance $Q_{dl}$ for the IDE topology; (**c**) charge-transfer resistance $R_{ct}$ for the HFE topology; (**d**) derived double-layer capacitance $Q_{dl}$ for the HFE topology. Data are shown for bare ITO and TiO_2_-coated electrodes with increasing TiO_2_ thickness.

The $R_{ct}$ values and the derived double-layer capacitance $Q_{dl}$ from the ${CPE}_{dl}$ element for both electrode topologies under UV illumination are plotted in Figure 16. For both electrode configurations, the bare ITO samples exhibit very large $R_{ct}$ values in the range of 10^7^–10^8^ Ω, indicating an almost completely blocking interface. In addition, these measurements show limited stability due to the extremely high impedance approaching the lower-frequency limit of the measurement window (10^5^–1 Hz).

In contrast, the TiO_2_-coated electrodes exhibit a pronounced collapse of $R_{ct}$, which scales with TiO_2_ layer thickness and reaches the lowest values at 0 mM Na^+^. For the IDE topology, the decrease corresponds to approximately 4.4 × 10^4^ Ω, 1.4 × 10^3^ Ω, and 2.5 × 10^2^ Ω for the 1×, 3×, and 5× TiO_2_ coatings, respectively. The evolution of the interfacial parameters can be related to the morphological and structural characteristics of the TiO_2_ layers (Figure 6). AFM analysis shows a transition from the relatively coarse ITO surface (RMS ≈ 4.8 nm, ~50 nm features) to a finer-grained TiO_2_ morphology (~20 nm) with reduced roughness (≈2.3–1.9 nm), indicating the formation of a continuous TiO_2_-covered interface. Despite the smoother surface, the electrochemical response becomes increasingly governed by TiO_2_. The decrease in $R_{ct}$ with increasing TiO_2_ thickness can be rationalized by the improved interfacial coverage and especially by the enhanced photoactivity associated with thicker layers [79,80]. At the same time, the decrease in the CPE exponent α (~0.96 to ~0.87; values listed in the Supplementary Materials Table S5) reflects increasing heterogeneity, consistent with the polycrystalline TiO_2_ structure (crystallite size ~14 nm from XRD) and the presence of grain boundaries affecting the observed interfacial behavior.

A similar but somewhat more pronounced decrease is observed for the HFE topology, yielding with 2.2 × 10^4^ Ω, 6.8 × 10^3^ Ω, and 2.2 × 10^2^ Ω for the corresponding coatings. This behavior can be attributed to the larger effective electrode surface area of the HFE topology, which is approximately 44% greater than that of the IDE configuration ($A_{ITO}^{HFE}>A_{ITO}^{IDE}$). Since the charge-transfer process occurs at the TiO_2_–electrolyte interface, and the equivalent circuit accounts for two identical interfaces in series, the extracted $R_{ct}$ values can be interpreted as representative of a single interface. Thus, the overall charge-transfer resistance is expected to scale inversely with the active TiO_2_ surface area in electrical contact with the conductive ITO layer. Accordingly, the decrease in $R_{ct}$ is accompanied by a proportional increase in the interfacial capacitance $Q_{dl}$.

As seen from the plots in Figure 16, the influence of Na^+^ concentration is relatively small and becomes noticeable only in the low-concentration range of 0–20 mM. This behavior is most likely associated with the increase in ionic strength, which compresses the electrical double layer and slightly modifies the interfacial polarization, resulting in a minor increase in $R_{ct}$ accompanied by a decrease in the magnitude of ${CPE}_{dl}$. Within the physiologically relevant range of 20–100 mM Na^+^, both $R_{ct}$ and ${CPE}_{dl}$ remain largely unaffected by Na^+^ concentration, indicating that these parameters are primarily governed by the organic components of the artificial sweat rather than by variations in electrolyte ionic strength.

#### 3.3.3. Effect of Organic Content

To confirm that the organic components of artificial sweat are responsible for the variations observed in the interfacial charge-transfer resistance ($R_{ct}$), additional electrochemical impedance spectroscopy (EIS) experiments were performed in which the concentrations of the two main organic constituents, lactic acid (LA) and urea, were systematically varied. The LA concentration was varied in the range of 2.5–30 mM, while the urea concentration was varied between 5 and 50 mM. All measurements were carried out under UV illumination, with the Na^+^ concentration fixed at 50 mM, corresponding to the midpoint of the range investigated in Section 3.3.2. In this way, the ionic strength of the electrolyte was maintained approximately constant while isolating the influence of the organic species.

**Figure 17:**
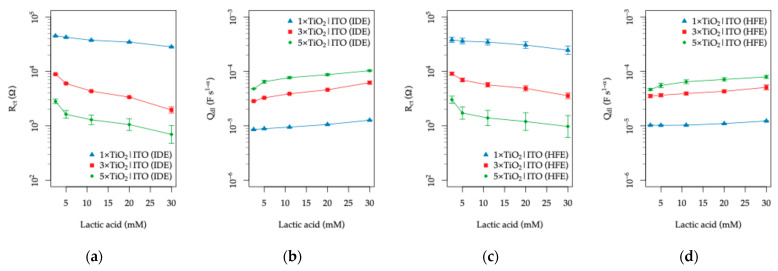
Dependence of the fitted interfacial charge-transfer parameters on the lactic acid (LA) concentration under UV illumination for artificial sweat solutions (50 mM Na^+^, 17 mM urea, 5 mM background K^+^): (**a**) charge-transfer resistance $R_{ct}$ for the IDE topology; (**b**) derived double-layer capacitance $Q_{dl}$ for the IDE topology; (**c**) charge-transfer resistance $R_{ct}$ for the HFE topology; (**d**) derived double-layer capacitance $Q_{dl}$ for the HFE topology.

Starting with the response to varied LA concentrations, the complete sets of Nyquist and Bode spectra, together with the fitted equivalent circuit parameters, are provided in the Supplementary Materials (Figures S8 and S9 and Table S6). The functional dependence of the fitted charge-transfer resistance ($R_{ct}$) and the double-layer capacitance parameter ($Q_{dl}$) on the LA concentration is summarized in Figure 17 for both IDE and HFE topologies.

For both electrode topologies, an observable decrease in $R_{ct}$ is detected with increasing LA concentration, accompanied by a minor increase in the double-layer capacitance. The previously discussed trend of lower $R_{ct}$ values for larger TiO_2_ thicknesses is preserved across the entire concentration range. Among the investigated electrodes, the 3×TiO_2_|ITO configuration shows the most pronounced response for both IDE and HFE topologies, indicating an optimal balance between the active semiconductor surface area and the effective transport of photogenerated charge carriers.

The decrease in $R_{ct}$ with increasing LA concentration can be attributed to the electroactive nature of lactic acid. Under UV illumination, the TiO_2_ layer generates electron–hole pairs, and the presence of oxidizable organic molecules such as LA facilitates interfacial charge transfer by acting as a hole scavenger. This process suppresses electron–hole recombination and promotes the participation of photogenerated holes in surface oxidation reactions, effectively lowering the interfacial charge-transfer resistance and increasing the availability of charge carriers within the TiO_2_|ITO electrode stack.

**Figure 18:**
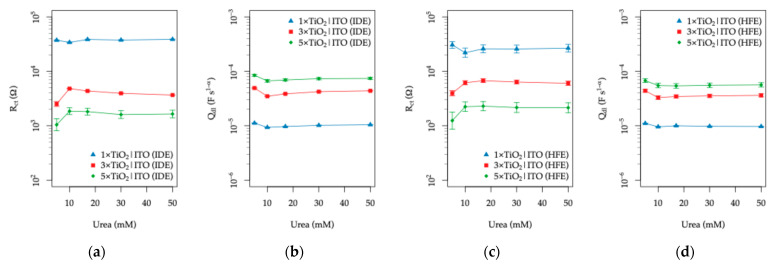
Dependence of the fitted interfacial charge-transfer parameters on the urea concentration under UV illumination for artificial sweat solutions (50 mM Na^+^, 11 mM lactic acid, 5 mM background K^+^): (**a**) charge-transfer resistance $R_{ct}$ for the IDE topology; (**b**) derived double-layer capacitance $Q_{dl}$ for the IDE topology; (**c**) charge-transfer resistance $R_{ct}$ for the HFE topology; (**d**) derived double-layer capacitance $Q_{dl}$ for the HFE topology.

In contrast, varying the urea concentration results in a negligible electrochemical response. The corresponding EIS spectra and fitted parameters are provided in the Supplementary Materials (Figures S10 and S11 and Table S7), while the extracted $R_{ct}$ and $Q_{dl}$ values are plotted in Figure 18.

Apart from a slight increase in $R_{ct}$ observed between 5 and 10 mM urea, which may be associated with weak adsorption or surface-site occupation by urea molecules, no systematic dependence of either $R_{ct}$ or $Q_{dl}$ on the urea concentration is observed for any of the electrode configurations. This behavior is consistent with the relatively low electrochemical activity of urea under the applied experimental conditions.

The differential response of the TiO_2_|ITO sensors toward the two chemical species can be explained by their distinct photocatalytic activity and interaction with the TiO_2_ surface. Lactic acid readily adsorbs on the TiO_2_ surface [81] and undergoes photocatalytic oxidation by photogenerated holes, acting as an efficient hole scavenger [82], and effectively suppressing electron–hole recombination. This process leads to the formation of intermediate oxidation products such as pyruvic or acetic acid [76].

In contrast, although urea can undergo photocatalytic oxidation on TiO_2_, its adsorption on the surface is weak [77], and its direct oxidation by photogenerated holes is relatively inefficient. Previous studies have shown that the photocatalytic degradation of urea is significantly enhanced only after surface modification with noble metals such as Pd or Pt [83]. Furthermore, urea oxidation on TiO_2_ proceeds predominantly through radical-mediated pathways rather than direct hole transfer, involving the transformation of amino groups and the formation of intermediates such as carbamic acid [84].

The contrasting responses toward LA and urea further support the interpretation that the dominant sensing mechanism of the TiO_2_|ITO electrodes under UV illumination is related to photo-assisted oxidation processes occurring at the semiconductor–electrolyte interface. While lactic acid can effectively participate in such reactions and modify the interfacial charge-transfer kinetics, urea behaves largely as an inert species within the investigated concentration range and therefore exerts only a minor influence on the electrochemical impedance response of the sensors.

The relative changes in $R_{ct}$ and $Q_{dl}$, listed in Table 3, were calculated as

(7) $${∆R}_{ct}=\frac{R_{ct}^{\prime \prime }-R_{ct}^{\prime }}{R_{ct}^{\prime }}\;\mathrm{and}\;{∆Q}_{dl}=\frac{Q_{dl}^{\prime \prime }-Q_{dl}^{\prime }}{Q_{dl}^{\prime }},$$

where $R_{ct}^{\prime }$, and $R_{ct}^{\prime \prime }$ and $Q_{dl}^{\prime }$, $Q_{dl}^{\prime \prime }$ are the charge-transfer resistance and double-layer capacitance at the beginning and end of the investigated concentration range, with reference to the first stable concentration point.

These relative changes further highlight the distinct response of the TiO_2_|ITO electrodes to lactic acid and urea. The IDE topology exhibits a pronounced decrease in $R_{ct}$ (up to 67%) accompanied by a significant increase in $Q_{dl}$ (up to 90%) with increasing lactic acid concentration, whereas the response to urea remains comparatively weak and non-systematic ($\left|∆\right|$ < 0.3). The TiO_2_|ITO electrodes with HFE topology exhibit similar trends, although with a moderately reduced response to lactic acid (approximately 20% lower in terms of ${∆R}_{ct}$ and ~45% lower in terms of ${∆Q}_{dl}$, on average).

The reduced modulation observed for the HFE topology under UV illumination can be explained by the different balance between resistive and capacitive contributions at the TiO_2_–electrolyte interface. While the HFE geometry provides a larger effective active area, resulting in lower baseline $R_{ct}$, it is also associated with higher interfacial capacitance. Therefore, relative changes $R_{ct}$ induced by lactic acid are partially damped when normalized to the initial state. In contrast, the IDE topology, characterized by higher baseline $R_{ct}$ and lower capacitance, exhibits larger relative modulation for comparable interfacial perturbations. Thus, while the HFE design enhances ionic conductivity sensing through geometric field effects, the UV-assisted interfacial response is governed by the interplay between charge-transfer resistance and capacitance, leading to a slightly reduced relative sensitivity.

In both cases, the TiO_2_|ITO structures with the thicker TiO_2_ overlayer (3× and 5×) yield larger relative changes. Thus, in the 5–30 mM LA range, the relative change in $R_{ct}$ corresponds to an approximate sensitivity of 1.3–2.7% mM^−1^ for the IDE topology and 1.3–1.9% mM^−1^ for the HFE topology, depending on the TiO_2_ layer thickness.

#### 3.3.4. Frequency-Resolved Response to Ionic and Organic Components and Dynamic Behavior of the TiO_2_|ITO Sensors

**Figure 19:**
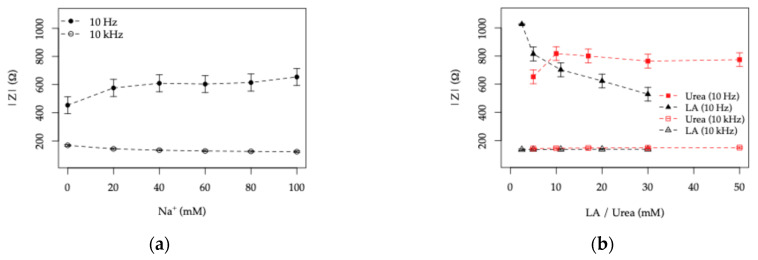

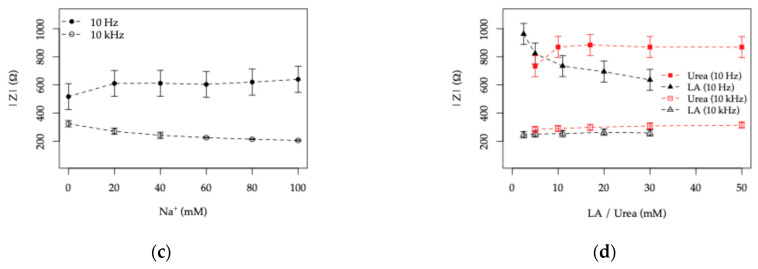
Dependence of the impedance magnitude $\left|Z\right|$ at 10 Hz and 10 kHz for 5×TiO_2_|ITO electrodes with different topologies in artificial sweat of varied composition: (**a**) IDE with Na^+^ varied in the 0–100 mM range; (**b**) IDE with lactic acid (2.5–30 mM) and urea (5–50 mM); (**c**) HFE with Na^+^ varied in the 0–100 mM range; (**d**) HFE with lactic acid (2.5–30 mM) and urea (5–50 mM).

To evaluate whether the impedimetric response can provide selective sensitivity toward ionic and organic components without relying on equivalent circuit fitting, the response of the 5×TiO_2_|ITO electrodes (highest TiO_2_ thickness) is presented in Figure 19 for both electrode topologies.

The impedance magnitude $\left|Z\right|$ was extracted at two representative frequencies, 10 Hz and 10 kHz. The low frequency was selected to probe interfacial processes while maintaining a reasonable acquisition rate, whereas the high frequency was chosen to enhance sensitivity to bulk electrolyte conductivity and minimize electrode polarization effects.

The dependence of $\left|Z\right|$ on Na^+^ concentration (Figure 19a,c) shows that variations in ionic content are predominantly reflected in the high-frequency (10 kHz) response, with a more pronounced sensitivity observed for the HFE topology. The low-frequency signal exhibits comparatively minor variations across the physiologically relevant range (20–80 mM), indicating good baseline stability under these conditions.

In contrast, when varying the organic content (Figure 19b,d), the high-frequency response remains largely unchanged. The increase in urea concentration produces only a small and non-systematic variation in the low-frequency signal, primarily at low concentrations (5–10 mM). Conversely, increasing lactic acid concentration results in a consistent decrease in the low frequency $\left|Z\right|$, in agreement with the trends observed from full EIS analysis and the extracted parameters summarized in Table 3.

These results indicate that the high-frequency impedance is primarily governed by electrolyte conductivity, while the low-frequency response under UV illumination is more sensitive to interfacial processes associated with lactic acid. This behavior suggests the possibility of distinguishing between ionic and organic contributions using frequency-resolved measurements, allowing for a partial signal separation.

A comparison of representative impedimetric lactate sensors reported in the literature is presented in Table 4, with emphasis on systems operating in sweat or sweat-like media and employing frequency-dependent signal extraction. Due to the limited number of impedance-based sensors for lactate detection, particularly under physiologically relevant conditions, the comparison includes both enzymatic and non-enzymatic approaches utilizing electrochemical impedance spectroscopy or closely related techniques. To enable a consistent evaluation, the sensor responses are expressed as relative signal changes per concentration unit, recalculated where necessary, while the corresponding frequency ranges are explicitly included to highlight differences in the probed interfacial and bulk processes. Notably, most reported systems rely on enzymatic reactions and monitor variations in charge-transfer resistance or equivalent parameters over a broad frequency spectrum, whereas the present TiO_2_|ITO system enables selective sensitivity through discrete frequency sampling. This frequency-resolved approach allows differentiation between ionic contributions, which are dominant at high frequency, and interfacial processes associated with lactic acid at low frequency, as demonstrated above.

**Figure 20:**
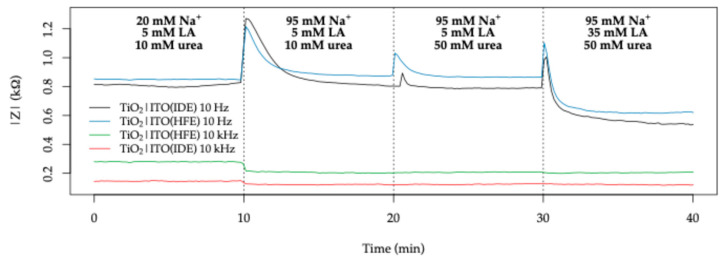
Time-resolved impedance response ($\left|Z\right|$ at 10 Hz and 10 kHz) of 5×TiO_2_|ITO electrodes with IDE and HFE topologies under continuous UV illumination during stepwise changes in artificial sweat composition. Sequential increases in Na^+^, urea, and lactic acid concentrations demonstrate stable operation, reproducible transient behavior, and distinct frequency-dependent sensitivity to ionic and organic components.

To further assess sensor stability and dynamic response under continuous operation, time-resolved $\left|Z\right|$ measurements were performed at 10 Hz and 10 kHz under constant UV illumination. The sensors were initially equilibrated in 0.5 mL artificial sweat (20 mM Na^+^, 5 mM lactic acid, 10 mM urea, 5 mM K^+^), followed by stepwise additions of concentrated solutions over three 10 min intervals, increasing sequentially the Na^+^ concentration to 95 mM, urea to 50 mM, and lactic acid to 35 mM. The results are shown in Figure 20.

Both electrode configurations exhibit stable operation over the full duration of the experiment (≈40 min), with reproducible transient responses following each compositional change and no evidence of signal degradation under continuous UV illumination. At high frequency (10 kHz), a clear step decrease in $\left|Z\right|$ is observed upon increasing Na^+^ concentration, which is consistent with enhanced electrolyte conductivity and in agreement with the steady-state measurements.

At low frequency (10 Hz), transient increases in $\left|Z\right|$ are observed immediately after each addition, followed by stabilization within approximately 3–5 min. The increase in urea concentration produces only a minor change in the stabilized signal, whereas the subsequent increase in lactic acid concentration results in a distinct shift in the low-frequency baseline. This behavior is consistent with the steady-state trends and supports the assignment of the low-frequency response to interfacial processes involving lactic acid under photo-assisted conditions.

The transient overshoots observed after each addition are attributed to mixing and local concentration gradients within the cell. Importantly, the recovery to stable baseline values and the absence of drift over time indicate that neither photocorrosion of the TiO_2_ layer nor significant surface fouling by reaction products occurs within the investigated time scale.

## 4. Conclusions

In summary, TiO_2_|ITO sensors were successfully fabricated on patterned ITO substrates in two configurations: a classical interdigitated electrode array (IDE) and a Hilbert fractal electrode (HFE) geometry. The effects of electrode topology and TiO_2_ layer thickness were investigated in artificial sweat with varied ionic content (0–100 mM Na^+^) and organic content (2.5–30 mM lactic acid and 5–50 mM urea). The impedimetric response of both configurations was studied by electrochemical impedance spectroscopy in darkness and under UV illumination. In darkness, all electrodes tracked changes in Na^+^ concentration, reflecting the ionic conductivity of the solutions. A strong effect of electrode topology was observed, with the HFE pattern exhibiting up to 71% higher impedance due to its increased perimeter density and non-uniform electric field distribution, as supported by simulations. At the same time, the HFE design showed approximately 2.5× higher modulation with respect to changes in ionic conductivity.

UV illumination did not significantly affect high-frequency response but activated a low-frequency contribution associated with the photocatalytic activity of the TiO_2_ layer. This response is governed by the interaction with organic components of the sweat, enabling their detection. Lactic acid produces a pronounced response in comparison to urea. The TiO_2_ thickness strongly influences this low-frequency behavior. The HFE topology exhibits a 20–45% lower modulation in the UV-assisted response (in terms of resistance and capacitance), while also exhibiting reduced relative sensitivity to urea due to the overall lower modulation of the UV-assisted interfacial response.

Overall, the results demonstrate that TiO_2_|ITO sensors enable frequency-dependent response to ionic conductivity and organic content, with high-frequency signals predominantly governed by electrolyte content and low-frequency signals under UV illumination influenced by interfacial processes involving lactic acid. These findings suggest that such systems may support the differentiation of ionic and organic contributions through frequency-resolved measurements, providing a promising basis for the development of photo-assisted, multi-parameter sweat sensors. Future work should focus on the implementation on flexible substrates and further optimization of the sensing layer, taking advantage of the low toxicity and photocatalytic self-cleaning potential of TiO_2_.

## Figures and Tables

**Table 1:** Geometrical parameters of the interdigitated electrode (IDE) and Hilbert fractal electrode (HFE) patterned ITO supports derived from the photomask design.

Parameter	Topology	
	IDE	HFE
Trace width	200 μm	300 μm
Opposing gap width	220 μm	150 μm
Interelectrode path length	210 mm	227 mm
Wetted conductive area	80.4 mm^2^	115.98 mm^2^

**Table 2:** Extracted fitting parameters for the $R_{s}\left(\sigma \right)$ dependence obtained using Equation (6). The parameters include the topology-dependent geometry factor (K) and the σ-independent base resistance term ($R_{0}$).

Electrode	IDE Topology				HFE Topology			
	K ($\Omega \;mS\;cm^{-1}$)		$R_{0}$ (Ω)		K ($\Omega \;mS\;cm^{-1}$)		$R_{0}$ (Ω)	
	Dark	UV	Dark	UV	Dark	UV	Dark	UV
Bare ITO	141.9	138.5	100.7	100.9	249.9	243.8	112.9	113.3
1×TiO_2_|ITO	102.5	100.1	107.7	107.8	254.9	252.1	137.3	136.5
3×TiO_2_|ITO	90.5	81.8	122.7	123.6	241.5	220.3	156.9	161.2
5×TiO_2_|ITO	105.8	83.4	112.2	116.5	244.1	214.9	195.2	194.8

**Table 3:** Relative changes in charge-transfer resistance (${∆R}_{ct}$) and double-layer capacitance (${∆Q}_{dl}$) for the TiO_2_|ITO electrodes with IDE and HFE topologies.

Electrode	IDE Topology				HFE Topology			
	Lactic Acid		Urea		Lactic Acid		Urea	
	${∆R}_{ct}$	${∆Q}_{dl}$	${∆R}_{ct}$	${∆Q}_{dl}$	${∆R}_{ct}$	${∆Q}_{dl}$	${∆R}_{ct}$	${∆Q}_{dl}$
1×TiO_2_|ITO	−0.333	0.432	0.137	0.120	−0.320	0.201	0.211	0.014
3×TiO_2_|ITO	−0.674	0.895	−0.237	0.259	−0.484	0.395	−0.021	0.100
5×TiO_2_|ITO	−0.573	0.596	−0.112	0.112	−0.433	0.438	−0.041	0.027

**Table 4:** Comparison of representative impedimetric lactate sensors operating in sweat-relevant media with emphasis on frequency-dependent response characteristics.

Material	Sensor Type	Medium	Frequency	Response/Linear Range	Ref.
TiO_2_|ITO (5×TiO_2_|ITO with IDE topology)	Non-enzymatic Impedimetric	Artificial sweat (pH = 6.5)	10 Hz	1.94% mM^−1^ (2.5–30 mM)	This work
TiO_2_|ITO (5×TiO_2_|ITO with HFE topology)				1.36% mM^−1^ (2.5–30 mM)	
Au/ZnO electrode functionalized with LOx	Enzymatic Impedimetric	Artificial sweat (pH 8)	10 Hz	8.3% mM^−1^ (0.1–100 mM)	[85]
GO/PANHS membrane, functionalized with LOx (Pd electrode)	Enzymatic Impedimetric	Synthetic sweat (pH = 4–8)	10 Hz	1.3% mM^−1^ * (1–100 mM)	[86]
Lactate-imprinted electropolymerized 3-APBA on screen-printed carbon support	Non-enzymatic Impedimetric	Synthetic sweat (pH = 6) human sweat	5 Hz–10 kHz	0.45% mM^−1^ * (3–100 mM)	[87]
LDH-modified screen-printed carbon	Enzymatic Impedimetric	PBS (pH = 7.4) Human sweat	20 Hz–20 MHz	0.28% mM^−1^ * (0.1–100 mM)	[88]

* Recalculated from the reported values.

## Data Availability

The original contributions presented in this study are included in the article/Supplementary Material. Further inquiries can be directed to the corresponding author.

## Supplementary Materials

The following supporting information can be downloaded at https://www.mdpi.com/article/10.3390/s26082365/s1; Table S1: List of artificial sweat compositions with varied components used in the electrode testing. The overall composition follows the EN1811 standard. The varied components are as follows: NaCl (Na^+^); DL-Lactic acid (DL-LA); Urea. KCl (K^+^) was fixed at 5 mM in all cases. The concentration of each tested series is listed, along the conductivity of the final solution. Figure S1. AFM images of the TiO_2_|ITO sensors: (**a**) microscopy image indicating the measurement location; (**b**) topology of 1×TiO_2_|ITO surface; (**c**) 3×TiO_2_|ITO surface; (**d**) 5×TiO_2_|ITO surface. Figure S2: Step height of the bare ITO and the TiO_2_|ITO sensors, prepared with one, three, and five dip-coated titania layers: (**a**) step height plots from the data, presented in Figure 6f–i, in the main text; (**b**) step height vs. the number of coating cycles. Table S2: Surface roughness of the bare ITO layer and the TiO_2_ dip-coated layers for TiO_2_|ITO sensors, prepared with one, three, and five dip-coated titania layers. Data for TiO_2_, deposited on the ITO surface and the etched glass substrate surface is presented, along with the corresponding ITO-glass step height. Figure S3: EIS measurements for the TiO_2_|ITO electrodes in distilled water under UV illumination: (**a**,**b**) Nyquist and corresponding Bode plot for the TiO_2_|ITO (IDE) topology; (**c**,**d**) Nyquist and corresponding Bode plot for the TiO_2_|ITO (HFE) topology. Table S3: EIS fitting parameters, obtained for response of the interdigitated electrode (IDE) and Hilbert fractal electrode (HFE) topologies with and without UV illumination in distilled water, presented in Figure 9 (main text) and S3. Figure S4: EIS data and fits for the Na^+^ response of the interdigitated electrode (IDE) topology without UV illumination for (**a**,**b**) bare ITO; (**c**,**d**) 1×TiO_2_|ITO; (**e**,**f**) 3×TiO_2_|ITO; and (**g**,**h**) 5×TiO_2_|ITO. Figure S5: EIS data and fits for the Na^+^ response of the Hilbert fractal electrode (HFE) topology without UV illumination for (**a**,**b**) bare ITO; (**c**,**d**) 1×TiO_2_|ITO; (**e**,**f**) 3×TiO_2_|ITO; and (**g**,**h**) 5×TiO_2_|ITO. Table S4: EIS fitting parameters, obtained for the Na^+^ response of the interdigitated electrode (IDE) and Hilbert fractal electrode (HFE) topologies without UV illumination, presented in Figures S4 and S5. Figure S6: EIS data and fits for the Na^+^ response of the interdigitated electrode (IDE) topology under UV illumination for (**a**,**b**) bare ITO; (**c**,**d**) 1×TiO_2_|ITO; (**e**,**f**) 3×TiO_2_|ITO; and (**g**,**h**) 5×TiO_2_|ITO. Figure S7: EIS data and fits for the Na^+^ response of the Hilbert fractal electrode (HFE) topology under UV illumination for (**a**,**b**) bare ITO; (**c**,**d**) 1×TiO_2_|ITO; (**e**,**f**) 3×TiO_2_|ITO; and (**g**,**h**) 5×TiO_2_|ITO. Table S5: EIS fitting parameters, obtained for the Na^+^ response of the interdigitated electrode (IDE) and Hilbert fractal electrode (HFE) topologies under UV illumination, presented in Figures S6 and S7. Figure S8: EIS data and fits for the lactic acid (LA) response of the interdigitated electrode (IDE) topology under UV illumination for (**a**,**b**) bare ITO; (**c**,**d**) 1×TiO_2_|ITO; (**e**,**f**) 3×TiO_2_|ITO; and (**g**,**h**) 5×TiO_2_|ITO. Figure S9: EIS data and fits for the lactic acid (LA) response of the Hilbert fractal electrode (HFE) topology under UV illumination for (**a**,**b**) bare ITO; (**c**,**d**) 1×TiO_2_|ITO; (**e**,**f**) 3×TiO_2_|ITO; and (**g**,**h**) 5×TiO_2_|ITO. Table S6: EIS fitting parameters, obtained for the lactic acid (LA) response of the interdigitated electrode (IDE) and Hilbert fractal electrode (HFE) topologies under UV illumination, presented in Figures S8 and S9. Figure S10: EIS data and fits for the urea response of the interdigitated electrode (IDE) topology under UV illumination for (**a**,**b**) bare ITO; (**c**,**d**) 1×TiO_2_|ITO; (**e**,**f**) 3×TiO_2_|ITO; and (**g**,**h**) 5×TiO_2_|ITO. Figure S11: EIS data and fits for the Urea response of the Hilbert fractal electrode (HFE) topology under UV illumination for (**a**,**b**) bare ITO; (**c**,**d**) 1×TiO_2_|ITO; (**e**,**f**) 3×TiO_2_|ITO; and (**g**,**h**) 5×TiO_2_|ITO. Table S7: EIS fitting parameters, obtained for the urea response of the interdigitated electrode (IDE) and Hilbert fractal electrode (HFE) topologies under UV illumination, presented in Figures S10 and S11.
