# Supplementary material for: Impedimetric Analysis of the Photocatalysis-Assisted Response of Patterned TiO2|ITO Electrodes Exposed to Artificial Sweat
Source: Sensors (Basel). 2026 Apr 11;26(8):2365. doi: 10.3390/s26082365 (PMC13120423; doi:10.3390/s26082365)
Supplement: Supplementary file 1 [file sensors-26-02365-s001.zip › sensors-4238469-supplementary.pdf]

Supplementary Materials

Impedimetric Analysis of the Photocatalysis-Assisted Response of Patterned TiO_2/ITO Electrodes Exposed to Artificial Sweat

Bozhidar I. Stefanov [1*], Boriana R. Tzaneva [1], Valentin M. Mateev [2] and Ivo T. Iliev [3]

1 Department of Chemistry, Faculty of Electronic Engineering and Technology, Technical University of Sofia, 8, Kliment Ohridski Blvd, 1000 Sofia, Bulgaria
2 Department of Electrical Apparatus, Faculty of Electrical Engineering, Technical University of Sofia, 8, Kliment Ohridski Blvd, 1000 Sofia, Bulgaria
3 Department of Electronics, Faculty of Electronic Engineering and Technology, Technical University of Sofia, 8, Kliment Ohridski Blvd, 1000 Sofia, Bulgaria
* Correspondence: b.stefanov@tu-sofia.bg (B.I.S.)

Table S1. List of artificial sweat compositions with varied components used in the electrode testing. The overall composition is according to the EN1811 standard. The varied components are as follows: NaCl (Na^+); DL-Lactic acid (LA); Urea. KCl (K^+) was fixed at 5 mM in all cases. The concentration of each tested series is listed, along the conductivity of the final solution.

Series	Na^+ (mM)	LA (mM)	Urea (mM)	K^+ (mM)	Conductivity (mS cm^{-1})
**Na^+ series (0 – 100 mM)**	0	11	17	5	1.58
	20				3.36
	40				5.18
	60				6.89
	80				8.52
	100				10.12
**DL-LA series (2.5 – 30 mM)**	50	2.5	17	5	5.37
		5			5.59
		11			6.04
		20			6.63
		30			7.37
**Urea series (5 – 50 mM)**	50	11	5	5	6.06
			10		5.98
			17		6.04
			30		6.03
			50		5.97

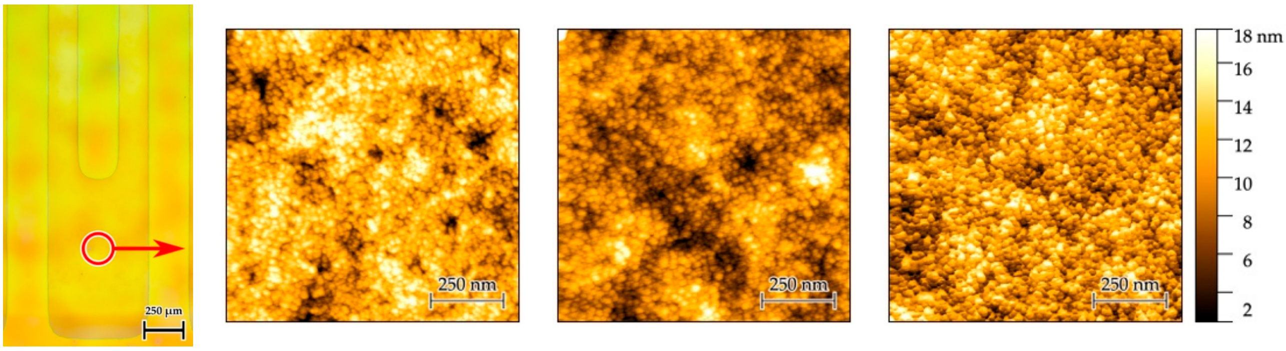

(**a**) (**b**) 1×TiO_2|ITO (**c**) 3×TiO_2|ITO (**d**) 5×TiO_2|ITO

Figure S1. AFM images of the TiO_2|ITO sensors: (**a**) microscopy image indicating the measurement location; (**b**) topology of 1×TiO_2|ITO surface; (**c**) 3×TiO_2|ITO surface; (**d**) 5×TiO_2|ITO surface.

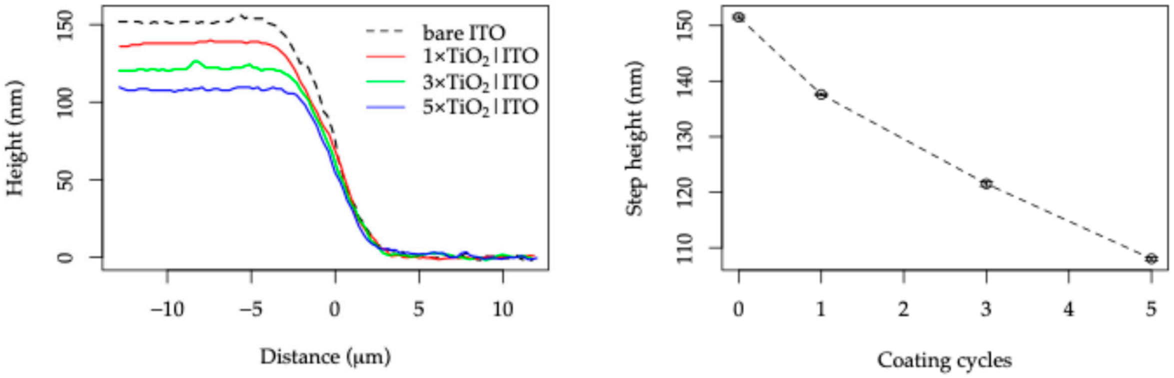

(**a**) 1×TiO_2|ITO (**b**) 3×TiO_2|ITO

Figure S2. Step-height of the bare ITO and the TiO_2|ITO sensors, prepared with 1, 3, and 5 dip-coated titania layers: (**a**) step-height plots from the data, presented in Figure 6f – 6i in the main text; (**b**) step-height vs. the number of coating cycles.

Table S2. Surface roughness of the bare ITO layer and the TiO_2 dip-coated layers for TiO_2|ITO sensors, prepared with 1, 3, and 5 dip-coated titania layers. Data for TiO_2, deposited on the ITO surface and the etched glass substrate surface is presented, along with the corresponding ITO-glass step height.

**Surface**	**ITO surface**		**Step height (nm)**	**Glass surface**	
	**R_a (nm)**	**R_q (nm)**		**R_a (nm)**	**R_q (nm)**
Bare ITO	3.799	4.827	151.5	-	-
1×TiO_2|ITO	1.825	2.273	137.6	1.959	2.464
3×TiO_2|ITO	1.485	1.871	121.5	1.496	1.943
5×TiO_2|ITO	1.523	1.906	108.0	1.525	1.897

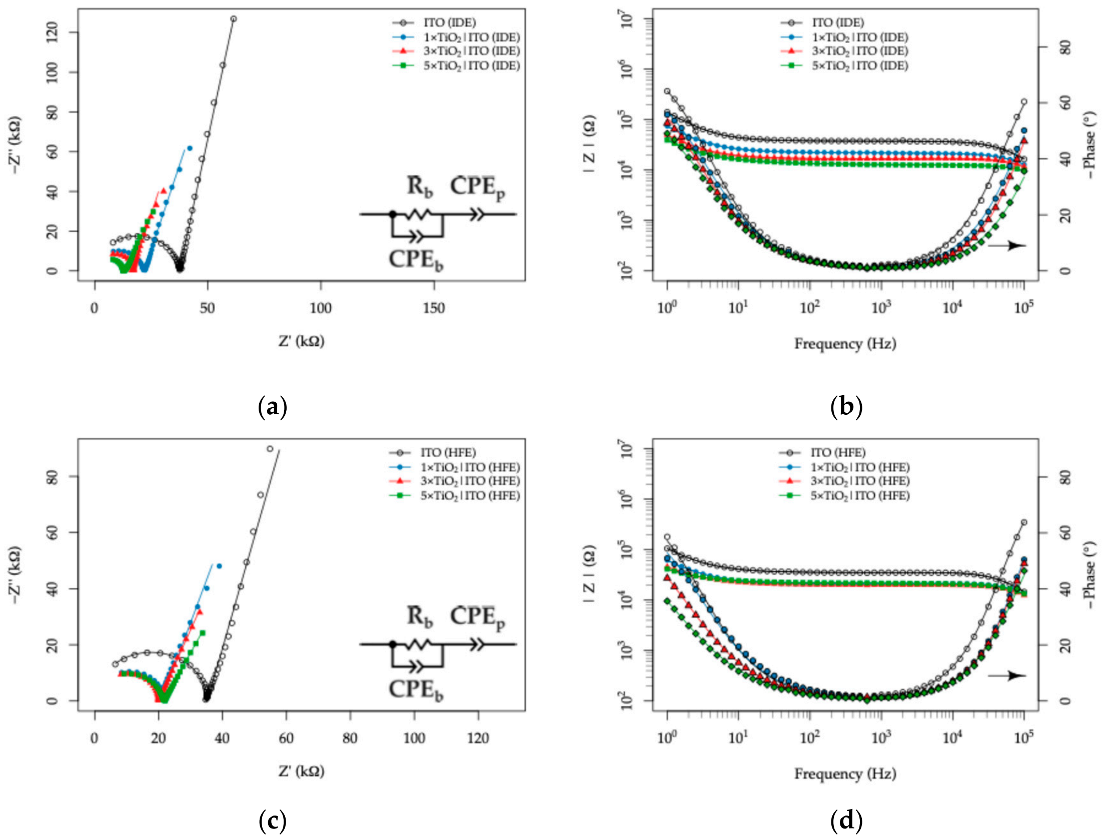

Figure S3. EIS measurements for the TiO_2|ITO electrodes in distilled water under UV illumination: (**a, b**) Nyquist and corresponding Bode plot for the TiO_2|ITO (IDE) topology; (**c, d**) Nyquist and corresponding Bode plot for the TiO_2|ITO (HFE) topology.

Table S3. EIS fitting parameters, obtained for response of the interdigitated electrode (IDE) and Hilbert fractal electrode (HFE) topologies with and without UV illumination in distilled water, presented in Figures 9 (main text) and Figure S3.

**Electrode**	**R_b (Ω)**	**CPE_b (F $s^{1-\alpha}$)**	**α**	**CPE_p (F $s^{1-\alpha}$)**	**α**
Bare ITO (IDE) (Dark)	3.87×10^{-4}	1.42×10^{-10}	0.958	1.57×10^{-6}	0.864
1×TiO_2|ITO (IDE) (Dark)	2.35×10^{-4}	1.08×10^{-10}	0.989	1.65×10^{-6}	0.899
3×TiO_2|ITO (IDE) (Dark)	2.48×10^{-4}	2.36×10^{-10}	0.931	1.68×10^{-6}	0.899
5×TiO_2|ITO (IDE) (Dark)	1.52×10^{-4}	8.97×10^{-10}	0.827	2.59×10^{-6}	0.797
Bare ITO (HFE) (Dark)	3.84×10^{-4}	1.61×10^{-10}	0.960	2.22×10^{-6}	0.834
1×TiO_2|ITO (HFE) (Dark)	2.67×10^{-4}	9.35×10^{-11}	1.000	2.59×10^{-6}	0.827
3×TiO_2|ITO (HFE) (Dark)	2.52×10^{-4}	2.43×10^{-10}	0.927	2.77×10^{-6}	0.820
5×TiO_2|ITO (HFE) (Dark)	2.11×10^{-4}	5.13×10^{-10}	0.861	2.80×10^{-6}	0.769
Bare ITO (IDE) (UV)	3.75×10^{-4}	1.39×10^{-10}	0.959	1.53×10^{-6}	0.881
1×TiO_2|ITO (IDE) (UV)	2.18×10^{-4}	1.92×10^{-10}	0.944	3.51×10^{-6}	0.818
3×TiO_2|ITO (IDE) (UV)	1.68×10^{-4}	9.81×10^{-11}	1.000	5.33×10^{-6}	0.822
5×TiO_2|ITO (IDE) (UV)	1.29×10^{-4}	2.75×10^{-10}	0.916	8.18×10^{-6}	0.749
Bare ITO (HFE) (UV)	3.52×10^{-4}	1.12×10^{-10}	0.989	2.30×10^{-6}	0.842
1×TiO_2|ITO (HFE) (UV)	2.08×10^{-4}	9.26×10^{-11}	1.000	4.51×10^{-6}	0.798
3×TiO_2|ITO (HFE) (UV)	1.99×10^{-4}	1.19×10^{-10}	0.983	7.36×10^{-6}	0.761
5×TiO_2|ITO (HFE) (UV)	2.15×10^{-4}	1.99×10^{-10}	0.933	1.02×10^{-5}	0.710

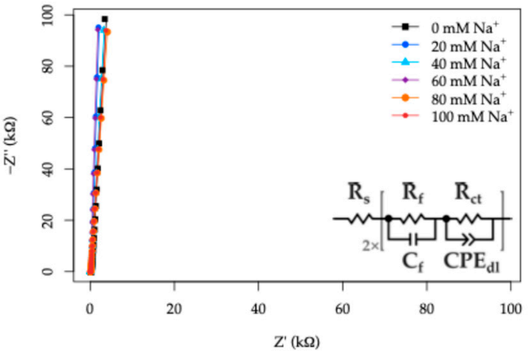

(**a**) Nyquist ITO (IDE), Dark

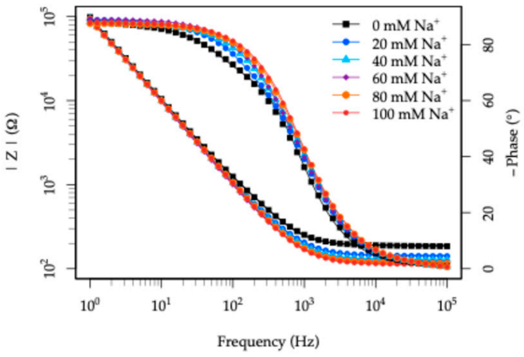

(**b**) Bode ITO (IDE), Dark

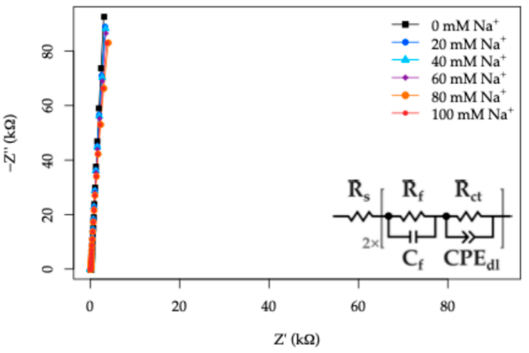

(**c**) Nyquist 1×TiO_2|ITO (IDE), Dark

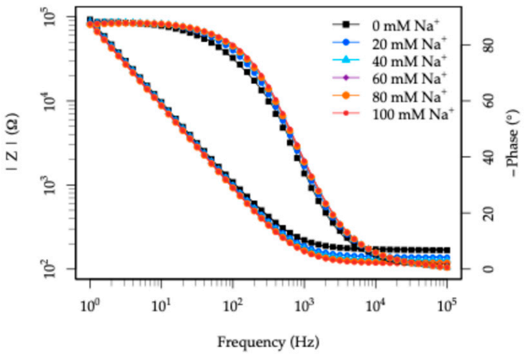

(**d**) Bode 1×TiO_2|ITO (IDE), Dark

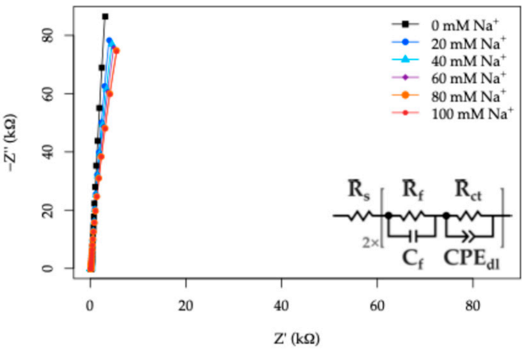

(**e**) Nyquist 3×TiO_2|ITO (IDE), Dark

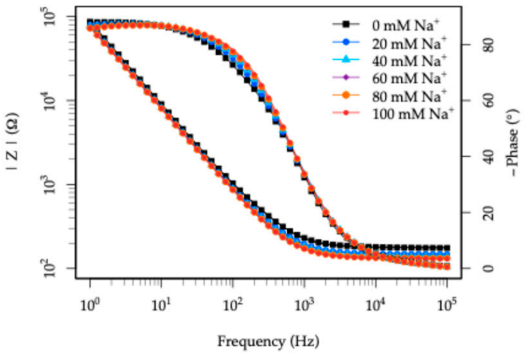

(**f**) Bode 3×TiO_2|ITO (IDE), Dark

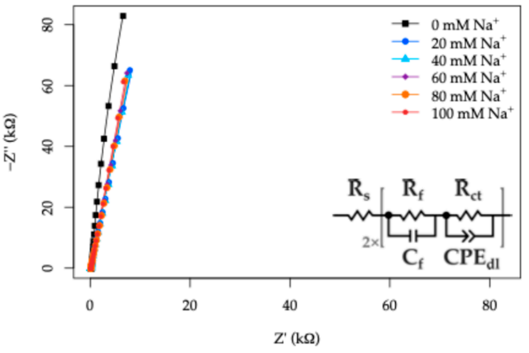

(**g**) Nyquist 5×TiO_2|ITO (IDE), Dark

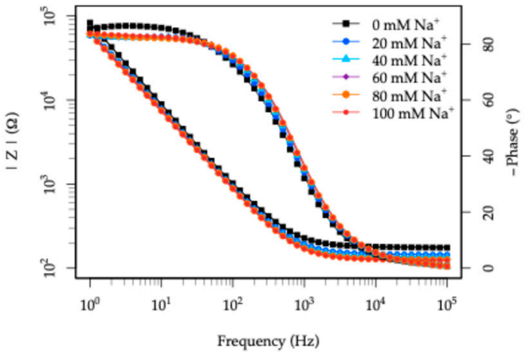

(**h**) Bode 5×TiO_2|ITO (IDE), Dark

Figure S4. EIS data and fits for the Na^+ response of the interdigitated electrode (IDE) topology without UV illumination for the: (**a, b**) bare ITO; (**c, d**) 1×TiO_2|ITO; (**e, f**) 3×TiO_2|ITO; and (**g, h**) 5×TiO_2|ITO.

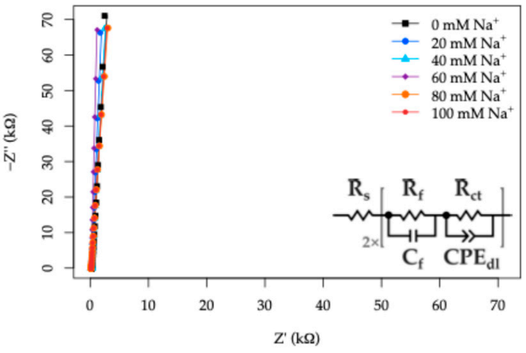

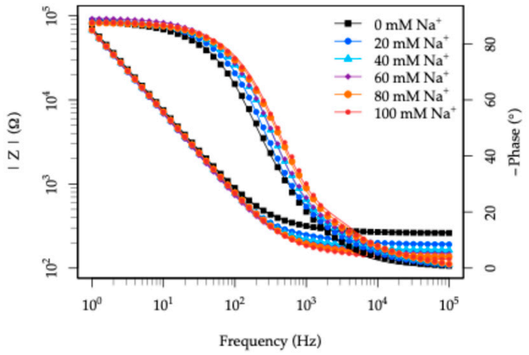

(**a**) Nyquist ITO (HFE), Dark (**b**) Bode ITO (HFE), Dark

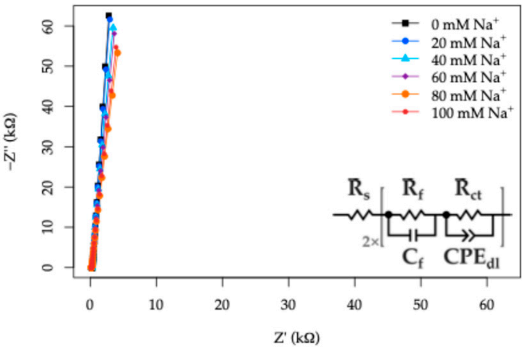

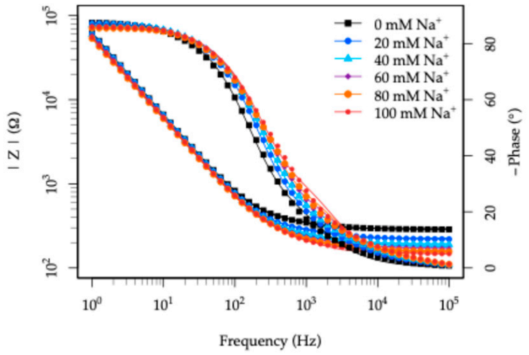

(**c**) Nyquist 1×TiO_2|ITO (HFE), Dark (**d**) Bode 1×TiO_2|ITO (HFE), Dark

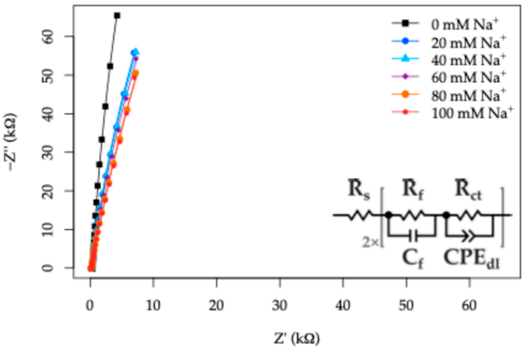

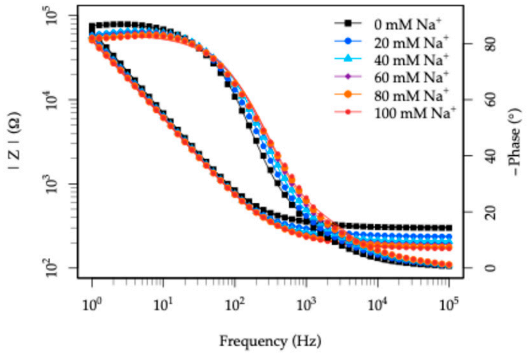

(**e**) Nyquist 3×TiO_2|ITO (HFE), Dark (**f**) Bode 3×TiO_2|ITO (HFE), Dark

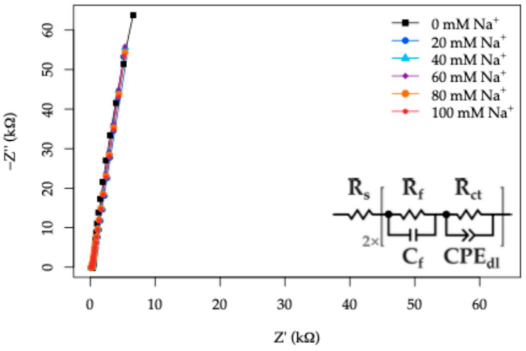

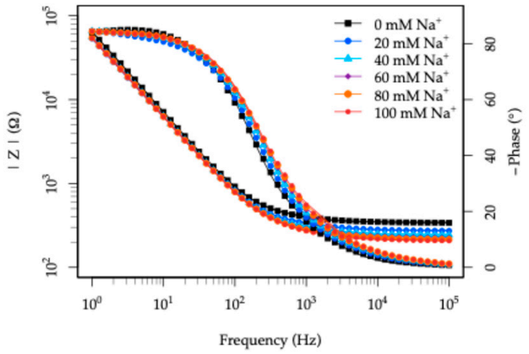

(**g**) Nyquist 5×TiO_2|ITO (HFE), Dark (**h**) Bode 5×TiO_2|ITO (HFE), Dark

Figure S5. EIS data and fits for the Na^+ response of the Hilbert fractal electrode (HFE) topology without UV illumination for the: (**a, b**) bare ITO; (**c, d**) 1×TiO_2|ITO; (**e, f**) 3×TiO_2|ITO; and (**g, h**) 5×TiO_2|ITO.

Table S4. EIS fitting parameters, obtained for the Na^+ response of the interdigitated electrode (IDE) and Hilbert fractal electrode (HFE) topologies without UV illumination, presented in Figures S4 & S5.

Topology	Na^+ (mM)	R_s (Ω)	R_f (Ω)	C_f (F)	R_{ct} (Ω)	CPE_{dl} (F $s^{1-\alpha}$)	CPE_{dl} α	χ^2
ITO (IDE) Dark	0	189.7	109.8	9.73×10^{-6}	1.23×10^{13}	3.37×10^{-6}	0.98	3.05×10^{-4}
	20	144.0	45.4	1.09×10^{-5}	6.59×10^{12}	3.42×10^{-6}	0.99	1.95×10^{-4}
	40	129.5	19.1	1.52×10^{-5}	3.24×10^{13}	3.52×10^{-6}	0.98	7.63×10^{-5}
	60	122.5	15.1	1.44×10^{-5}	1.61×10^{7}	3.43×10^{-6}	0.99	9.02×10^{-5}
	80	116.6	5.5	1.83×10^{-5}	1.26×10^{8}	3.57×10^{-6}	0.97	3.81×10^{-5}
	100	112.4	3.7	1.67×10^{-5}	4.76×10^{7}	3.53×10^{-6}	0.98	5.76×10^{-5}
1×TiO_2|ITO (IDE) Dark	0	171.5	48.4	1.60×10^{-5}	2.45×10^{7}	3.55×10^{-6}	0.98	2.03×10^{-4}
	20	139.6	17.9	1.86×10^{-5}	6.99×10^{6}	3.69×10^{-6}	0.98	9.39×10^{-5}
	40	128.6	10.8	1.99×10^{-5}	6.12×10^{6}	3.73×10^{-6}	0.98	5.36×10^{-5}
	60	122.1	7.4	2.13×10^{-5}	5.67×10^{6}	3.80×10^{-6}	0.98	4.25×10^{-5}
	80	119.2	8.2	1.93×10^{-5}	2.46×10^{6}	3.96×10^{-6}	0.98	5.25×10^{-5}
	100	115.9	6.5	2.13×10^{-5}	3.32×10^{6}	3.96×10^{-6}	0.98	5.00×10^{-5}
3×TiO_2|ITO (IDE) Dark	0	179.6	59.1	1.12×10^{-5}	8.11×10^{6}	3.79×10^{-6}	0.98	2.60×10^{-4}
	20	150.2	27.2	1.45×10^{-5}	2.82×10^{6}	4.21×10^{-6}	0.98	1.33×10^{-4}
	40	140.9	16.2	1.67×10^{-5}	1.92×10^{6}	4.29×10^{-6}	0.98	8.96×10^{-5}
	60	135.5	10.2	1.87×10^{-5}	1.57×10^{6}	4.36×10^{-6}	0.98	6.60×10^{-5}
	80	133.2	7.6	2.02×10^{-5}	1.20×10^{6}	4.42×10^{-6}	0.97	6.08×10^{-5}
	100	130.9	5.4	2.00×10^{-5}	1.24×10^{6}	4.45×10^{-6}	0.97	5.52×10^{-5}
5×TiO_2|ITO (IDE) Dark	0	178.9	44.1	1.32×10^{-5}	1.23×10^{6}	4.00×10^{-6}	0.97	1.73×10^{-4}
	20	144.4	1.1	9.00×10^{-6}	3.62×10^{6}	5.53×10^{-6}	0.93	2.77×10^{-4}
	40	132.7	<1	9.00×10^{-7}	2.90×10^{7}	5.71×10^{-6}	0.92	4.08×10^{-4}
	60	126.7	<1	2.32×10^{-6}	2.01×10^{7}	5.61×10^{-6}	0.93	3.70×10^{-4}
	80	124.1	<1	3.18×10^{-6}	1.32×10^{8}	5.88×10^{-6}	0.92	1.97×10^{-4}
	100	123.2	<1	9.82×10^{-6}	4.22×10^{9}	5.88×10^{-6}	0.93	3.88×10^{-5}
ITO (HFE) Dark	0	267.3	36.2	6.72×10^{-6}	1.47×10^{13}	4.66×10^{-6}	0.98	3.80×10^{-4}
	20	195.1	24.9	3.73×10^{-6}	7.92×10^{13}	4.94×10^{-6}	0.98	2.47×10^{-4}
	40	165.6	19.2	2.77×10^{-6}	7.23×10^{12}	4.92×10^{-6}	0.98	1.30×10^{-4}
	60	151.1	21.1	3.14×10^{-6}	1.35×10^{8}	4.82×10^{-6}	0.99	3.33×10^{-4}
	80	139.3	17.9	2.91×10^{-6}	5.48×10^{7}	4.93×10^{-6}	0.97	2.89×10^{-4}
	100	130.0	17.8	3.29×10^{-6}	1.69×10^{7}	4.92×10^{-6}	0.98	4.18×10^{-4}
1×TiO_2|ITO (HFE) Dark	0	293.0	39.6	5.07×10^{-6}	7.07×10^{12}	5.33×10^{-6}	0.98	3.36×10^{-4}
	20	225.1	32.1	3.46×10^{-6}	1.23×10^{12}	5.43×10^{-6}	0.97	3.68×10^{-4}
	40	194.3	30.6	3.38×10^{-6}	6.53×10^{6}	5.65×10^{-6}	0.97	5.23×10^{-4}
	60	175.3	30.1	3.76×10^{-6}	6.26×10^{6}	5.82×10^{-6}	0.97	6.98×10^{-4}
	80	162.1	30.1	4.15×10^{-6}	4.14×10^{6}	6.42×10^{-6}	0.96	7.99×10^{-4}
	100	152.7	32.5	4.85×10^{-6}	7.00×10^{6}	6.24×10^{-6}	0.96	9.92×10^{-4}
3×TiO_2|ITO (HFE) Dark	0	305.0	34.8	4.87×10^{-6}	1.18×10^{6}	5.01×10^{-6}	0.98	2.15×10^{-4}
	20	238.7	22.5	3.04×10^{-6}	5.50×10^{5}	6.04×10^{-6}	0.96	1.75×10^{-4}
	40	209.7	22.9	3.01×10^{-6}	5.05×10^{5}	6.01×10^{-6}	0.96	2.70×10^{-4}
	60	193.0	22.2	3.19×10^{-6}	5.40×10^{5}	6.28×10^{-6}	0.95	3.96×10^{-4}
	80	180.1	20.4	3.40×10^{-6}	6.36×10^{5}	6.90×10^{-6}	0.94	4.99×10^{-4}
	100	173.4	21.2	3.82×10^{-6}	7.53×10^{5}	7.13×10^{-6}	0.93	6.25×10^{-4}
5×TiO_2|ITO (HFE) Dark	0	345.3	34.6	4.92×10^{-6}	8.91×10^{5}	5.27×10^{-6}	0.96	1.95×10^{-4}
	20	277.6	28.4	4.12×10^{-6}	6.29×10^{13}	6.69×10^{-6}	0.94	2.44×10^{-4}
	40	247.3	26.8	3.38×10^{-6}	1.18×10^{7}	6.33×10^{-6}	0.94	2.42×10^{-4}
	60	231.6	28.1	3.83×10^{-6}	3.97×10^{6}	6.25×10^{-6}	0.95	3.57×10^{-4}
	80	220.2	28.5	4.00×10^{-6}	7.99×10^{6}	6.48×10^{-6}	0.94	4.11×10^{-4}
	100	212.0	28.5	4.30×10^{-6}	5.25×10^{10}	6.62×10^{-6}	0.94	4.14×10^{-4}

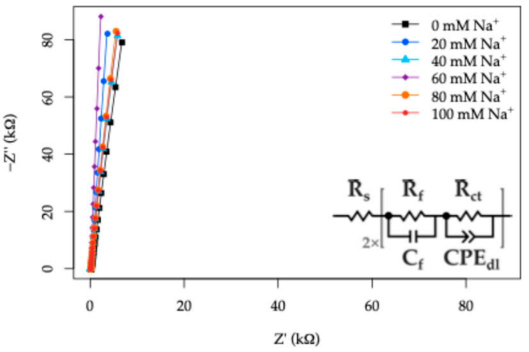

(**a**) Nyquist ITO (IDE), UV

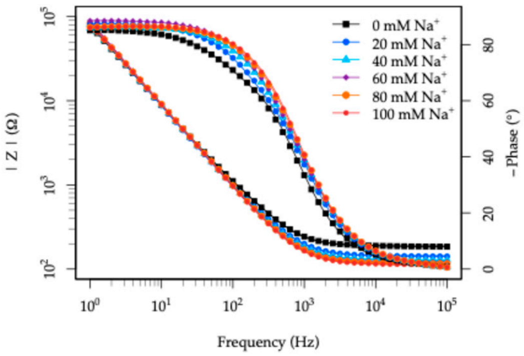

(**b**) Bode ITO (IDE), UV

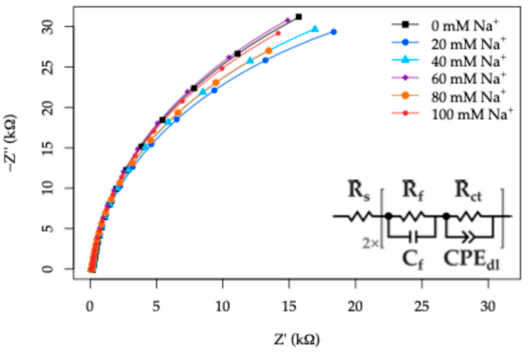

(**c**) Nyquist 1×TiO2|ITO (IDE), UV

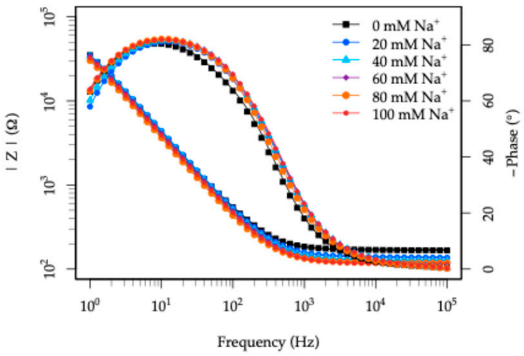

(**d**) Bode 1×TiO2|ITO (IDE), UV

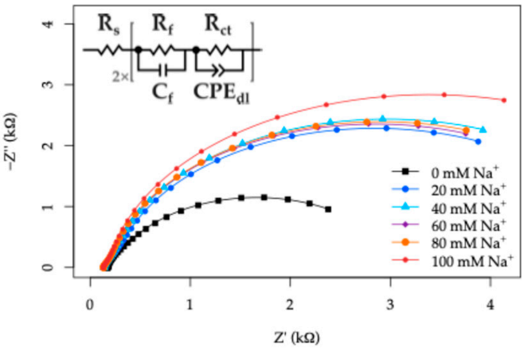

(**e**) Nyquist 3×TiO2|ITO (IDE), UV

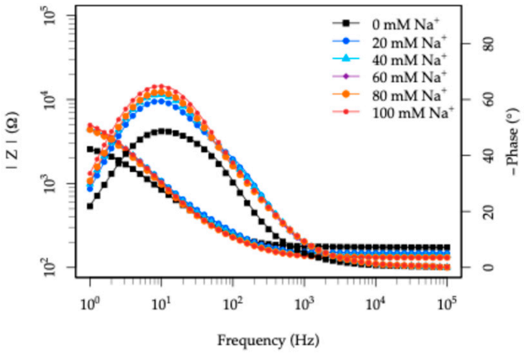

(**f**) Bode 3×TiO2|ITO (IDE), UV

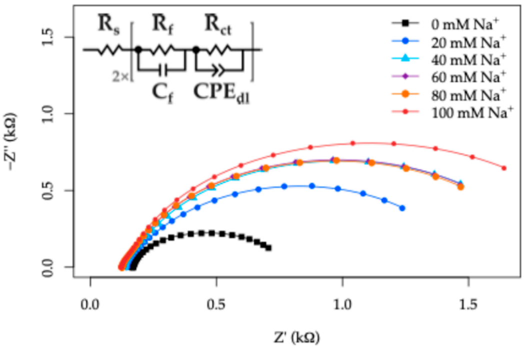

(**g**) Nyquist 5×TiO2|ITO (IDE), UV

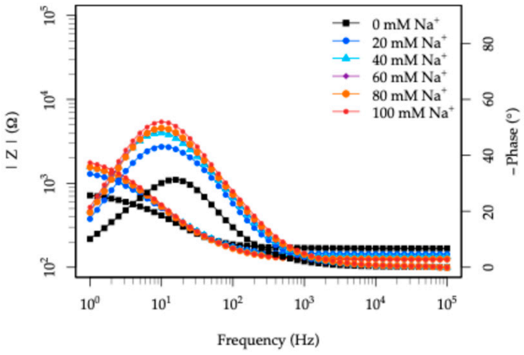

(**h**) Bode 5×TiO2|ITO (IDE), UV

Figure S6. EIS data and fits for the Na^+ response of the interdigitated electrode (IDE) topology under UV illumination for the: (**a, b**) bare ITO; (**c, d**) 1×TiO_2|ITO; (**e, f**) 3×TiO_2|ITO; and (**g, h**) 5×TiO_2|ITO.

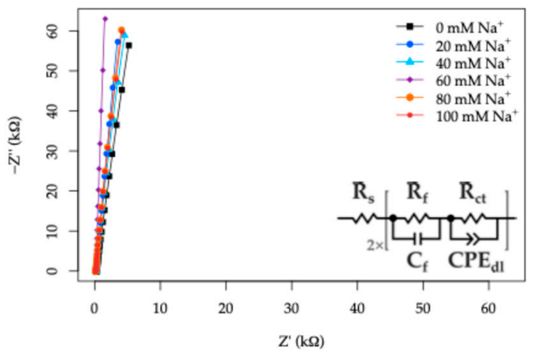

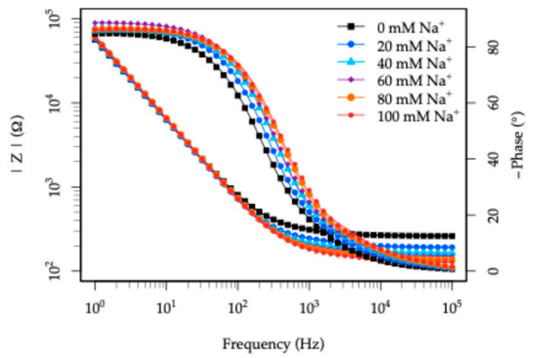

(**a**) Nyquist ITO (HFE), UV

(**b**) Bode ITO (HFE), UV

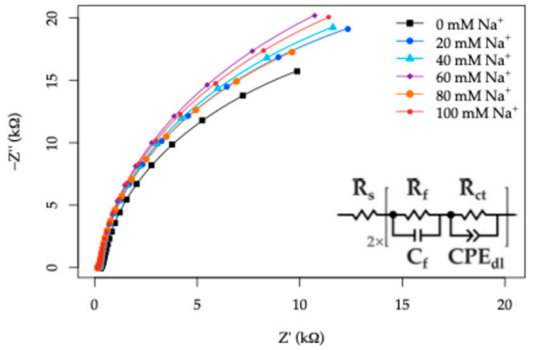

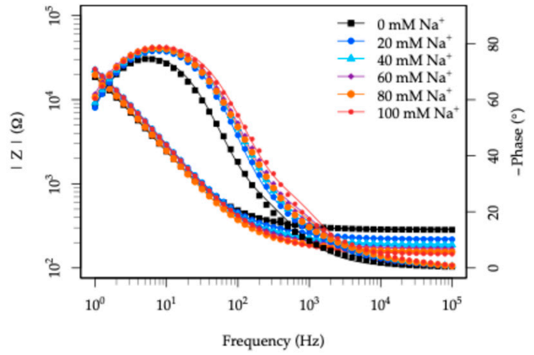

(**c**) Nyquist 1×TiO_2|ITO (HFE), UV

(**d**) Bode 1×TiO_2|ITO (HFE), UV

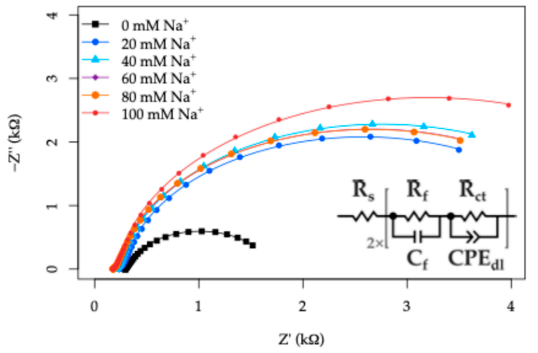

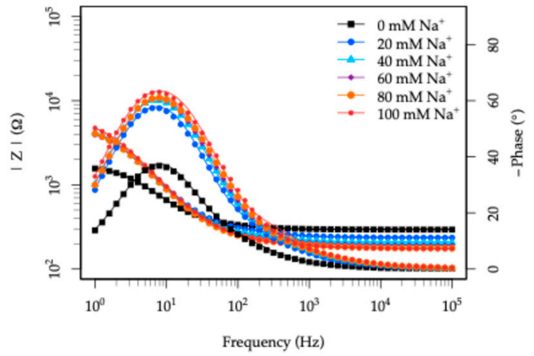

(**e**) Nyquist 3×TiO_2|ITO (HFE), UV

(**f**) Bode 3×TiO_2|ITO (HFE), UV

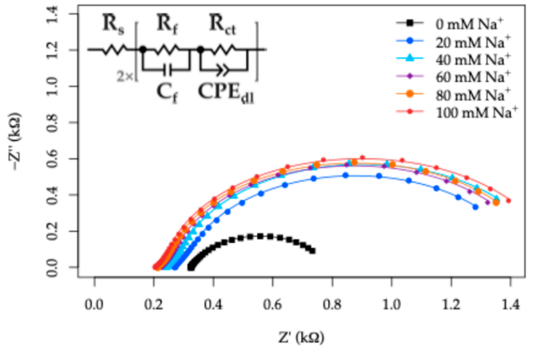

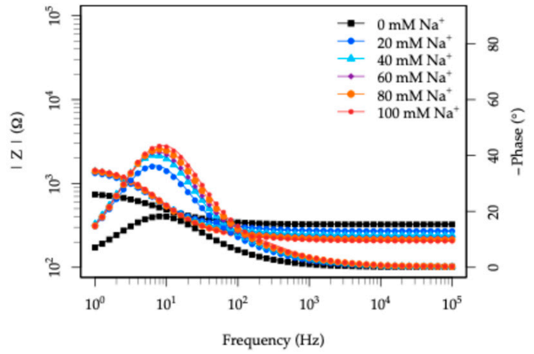

(**g**) Nyquist 5×TiO_2|ITO (HFE), UV

(**h**) Bode 5×TiO_2|ITO (HFE), UV

Figure S7. EIS data and fits for the Na^+ response of the Hilbert fractal electrode (HFE) topology under UV illumination for the: (**a, b**) bare ITO; (**c, d**) 1×TiO_2|ITO; (**e, f**) 3×TiO_2|ITO; and (**g, h**) 5×TiO_2|ITO.

Table S5. EIS fitting parameters, obtained for the Na^+ response of the interdigitated electrode (IDE) and Hilbert fractal electrode (HFE) topologies under UV illumination, presented in Figures S6 & S7.

Topology	Na^+ (mM)	R_s (Ω)	R_f (Ω)	C_f (F)	R_{ct} (Ω)	CPE_{dl} (F $s^{1-\alpha}$)	α	χ^2
ITO (IDE) UV	0	187.8	71.3	1.33×10^{-5}	6.72×10^{6}	4.38×10^{-6}	0.95	1.07×10^{-4}
	20	143.5	37.4	1.26×10^{-5}	1.02×10^{8}	4.06×10^{-6}	0.97	1.25×10^{-4}
	40	128.8	10.3	1.83×10^{-5}	3.56×10^{6}	4.16×10^{-6}	0.96	4.14×10^{-5}
	60	122.5	15.2	1.53×10^{-5}	1.75×10^{7}	3.70×10^{-6}	0.99	7.06×10^{-5}
	80	116.5	3.7	1.97×10^{-5}	4.21×10^{6}	4.07×10^{-6}	0.97	4.90×10^{-5}
	100	112.3	2.0	1.42×10^{-5}	2.60×10^{6}	4.09×10^{-6}	0.97	8.26×10^{-5}
1×TiO_2|ITO (IDE) UV	0	170.4	45.8	3.46×10^{-5}	4.37×10^{4}	8.74×10^{-6}	0.97	2.07×10^{-4}
	20	139.1	20.3	3.53×10^{-5}	3.62×10^{4}	8.43×10^{-6}	0.96	1.19×10^{-4}
	40	128.3	14.7	4.12×10^{-5}	3.85×10^{4}	8.73×10^{-6}	0.96	9.52×10^{-5}
	60	122.1	12.2	4.58×10^{-5}	4.52×10^{4}	9.05×10^{-6}	0.96	7.92×10^{-5}
	80	119.1	11.5	5.24×10^{-5}	3.84×10^{4}	1.01×10^{-5}	0.97	7.24×10^{-5}
	100	116.4	16.3	5.59×10^{-5}	4.25×10^{4}	9.53×10^{-6}	0.96	1.39×10^{-4}
3×TiO_2|ITO (IDE) UV	0	174.9	46.9	9.51×10^{-5}	1.40×10^{3}	7.46×10^{-5}	0.87	4.23×10^{-5}
	20	149.0	52.1	3.74×10^{-5}	2.59×10^{3}	4.45×10^{-5}	0.92	7.32×10^{-5}
	40	140.2	38.5	3.85×10^{-5}	2.75×10^{3}	4.45×10^{-5}	0.92	5.80×10^{-5}
	60	135.2	29.0	4.33×10^{-5}	2.66×10^{3}	4.66×10^{-5}	0.92	4.92×10^{-5}
	80	132.8	26.8	3.91×10^{-5}	2.72×10^{3}	4.73×10^{-5}	0.92	6.34×10^{-5}
	100	131.2	23.6	4.08×10^{-5}	3.21×10^{3}	4.40×10^{-5}	0.92	7.40×10^{-5}
5×TiO_2|ITO (IDE) UV	0	168.3	58.4	2.18×10^{-4}	2.47×10^{2}	2.87×10^{-4}	0.82	9.01×10^{-6}
	20	143.4	21.4	2.00×10^{-4}	6.47×10^{2}	1.30×10^{-4}	0.87	1.44×10^{-5}
	40	134.2	19.7	1.43×10^{-4}	8.22×10^{2}	1.10×10^{-4}	0.89	2.06×10^{-5}
	60	128.3	16.5	1.40×10^{-4}	8.20×10^{2}	1.05×10^{-4}	0.90	1.11×10^{-5}
	80	125.5	14.9	1.28×10^{-4}	8.17×10^{2}	1.03×10^{-4}	0.89	1.08×10^{-5}
	100	123.3	13.1	1.21×10^{-4}	9.59×10^{2}	9.71×10^{-4}	0.89	7.94×10^{-5}
ITO (HFE) UV	0	263.8	22.6	5.76×10^{-6}	3.12×10^{6}	6.14×10^{-6}	0.95	9.91×10^{-5}
	20	193.8	20.1	3.50×10^{-6}	8.05×10^{6}	5.89×10^{-6}	0.97	1.05×10^{-4}
	40	165.1	16.3	2.67×10^{-6}	1.77×10^{6}	5.74×10^{-6}	0.96	9.92×10^{-5}
	60	151.1	20.4	3.19×10^{-7}	7.96×10^{6}	5.17×10^{-6}	0.99	3.15×10^{-4}
	80	139.0	16.5	2.88×10^{-6}	2.94×10^{6}	5.60×10^{-6}	0.97	2.24×10^{-5}
	100	129.4	15.9	3.15×10^{-6}	2.18×10^{6}	5.64×10^{-6}	0.97	3.17×10^{-5}
1×TiO_2|ITO (HFE) UV	0	290.4	45.3	1.38×10^{-5}	2.04×10^{4}	1.63×10^{-5}	0.95	4.68×10^{-4}
	20	223.5	32.3	7.29×10^{-6}	2.37×10^{4}	1.30×10^{-5}	0.96	4.38×10^{-4}
	40	192.9	30.6	7.06×10^{-6}	2.48×10^{4}	1.33×10^{-5}	0.96	5.96×10^{-4}
	60	174.3	31.0	8.06×10^{-6}	2.86×10^{4}	1.35×10^{-5}	0.96	8.42×10^{-4}
	80	160.3	27.5	8.83×10^{-6}	2.35×10^{4}	1.55×10^{-5}	0.96	8.81×10^{-4}
	100	152.2	30.8	9.22×10^{-6}	2.70×10^{4}	1.32×10^{-5}	0.95	1.09×10^{-4}
3×TiO_2|ITO (HFE) UV	0	295.5	17.8	6.37×10^{-5}	6.81×10^{2}	9.36×10^{-5}	0.91	6.75×10^{-5}
	20	238.6	25.3	2.47×10^{-5}	2.29×10^{3}	4.93×10^{-5}	0.94	2.26×10^{-4}
	40	209.1	23.1	2.09×10^{-5}	2.50×10^{3}	4.81×10^{-5}	0.94	2.75×10^{-4}
	60	193.2	20.8	2.05×10^{-5}	2.42×10^{3}	4.94×10^{-5}	0.94	3.03×10^{-4}
	80	180.1	20.0	1.99×10^{-5}	2.43×10^{3}	4.94×10^{-5}	0.94	3.96×10^{-4}
	100	177.8	21.1	2.18×10^{-5}	2.98×10^{3}	4.52×10^{-5}	0.94	5.49×10^{-4}
5×TiO_2|ITO (HFE) UV	0	324.8	4.4	3.09×10^{-4}	2.24×10^{2}	2.49×10^{-4}	0.84	4.74×10^{-6}
	20	271.7	18.1	5.88×10^{-5}	5.69×10^{2}	1.18×10^{-4}	0.92	6.70×10^{-5}
	40	243.5	17.2	4.74×10^{-5}	6.28×10^{2}	1.05×10^{-4}	0.94	7.49×10^{-5}
	60	226.4	16.0	4.41×10^{-5}	6.16×10^{2}	1.02×10^{-4}	0.94	8.89×10^{-5}
	80	214.9	15.0	4.24×10^{-5}	6.33×10^{2}	9.61×10^{-4}	0.94	9.61×10^{-5}
	100	206.4	14.3	4.16×10^{-5}	6.62×10^{2}	9.11×10^{-4}	0.94	1.18×10^{-4}

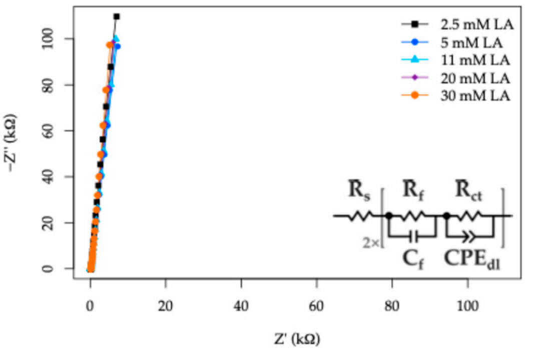

(**a**) Nyquist ITO (IDE), UV

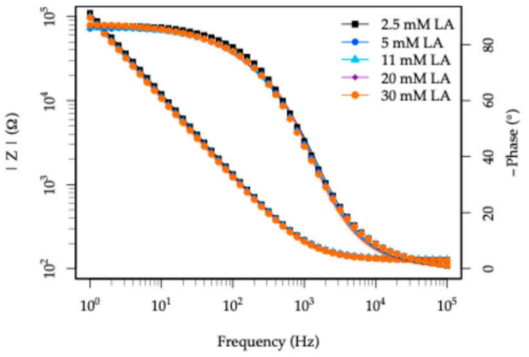

(**b**) Bode ITO (IDE), UV

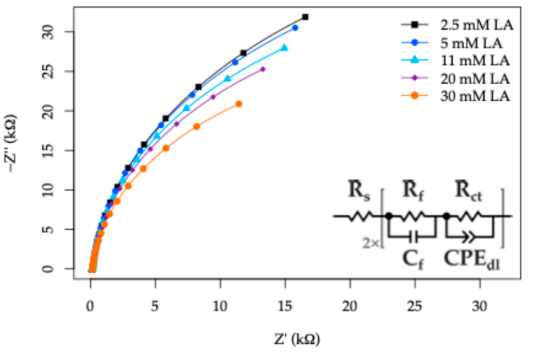

(**c**) Nyquist 1×TiO_2|ITO (IDE), UV

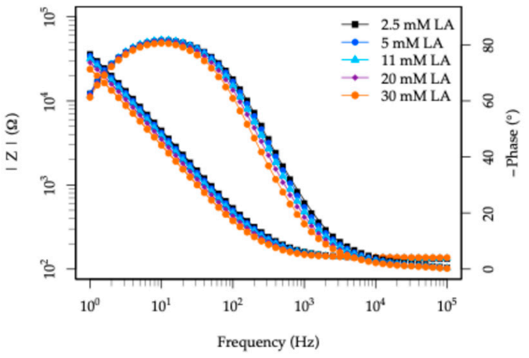

(**d**) Bode 1×TiO_2|ITO (IDE), UV

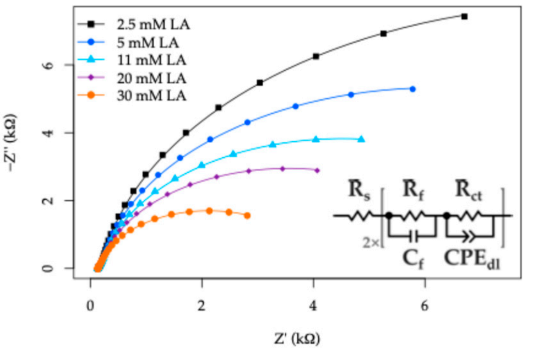

(**e**) Nyquist 3×TiO_2|ITO (IDE), UV

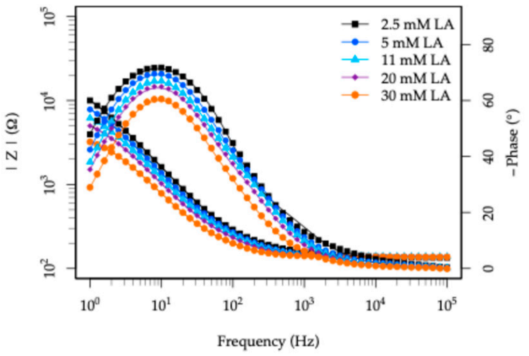

(**f**) Bode 3×TiO_2|ITO (IDE), UV

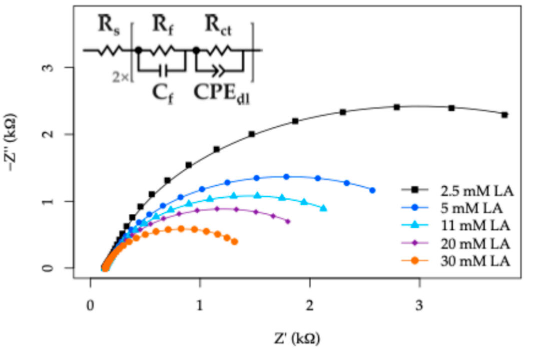

(**g**) Nyquist 5×TiO_2|ITO (IDE), UV

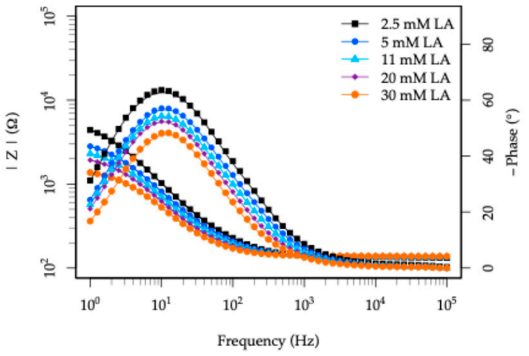

(**h**) Bode 5×TiO_2|ITO (IDE), UV

Figure S8. EIS data and fits for the Lactic acid (LA) response of the interdigitated electrode (IDE) topology under UV illumination for the: (**a, b**) bare ITO; (**c, d**) 1×TiO_2|ITO; (**e, f**) 3×TiO_2|ITO; and (**g, h**) 5×TiO_2|ITO.

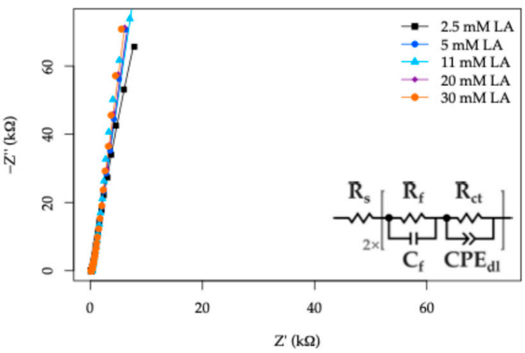

(**a**) Nyquist ITO (HFE), UV

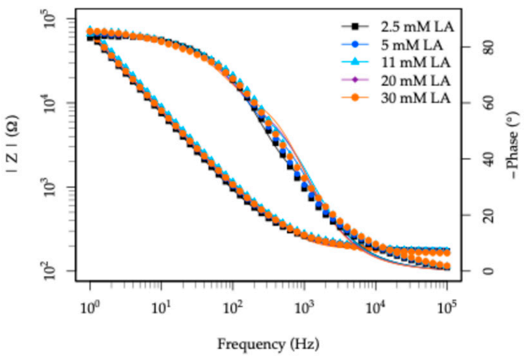

(**b**) Bode ITO (HFE), UV

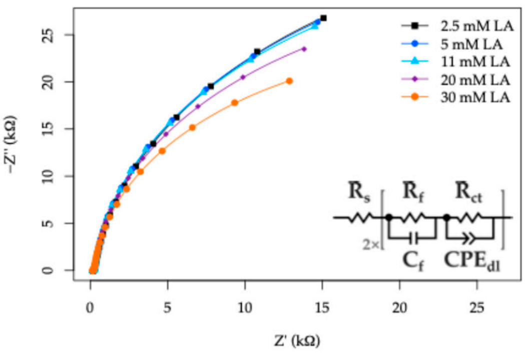

(**c**) Nyquist 1×TiO2 | ITO (HFE), UV

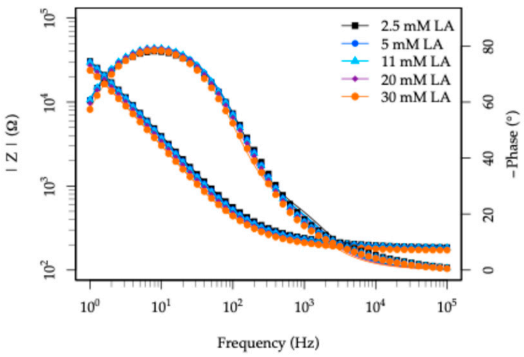

(**d**) Bode 1×TiO2 | ITO (HFE), UV

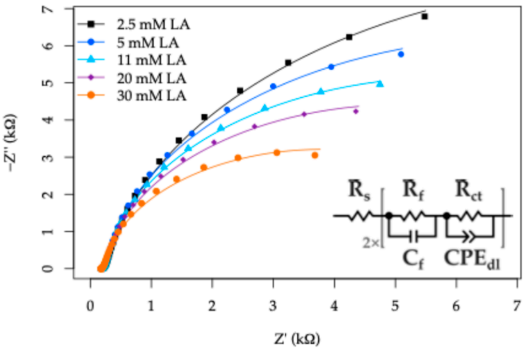

(**e**) Nyquist 3×TiO2 | ITO (HFE), UV

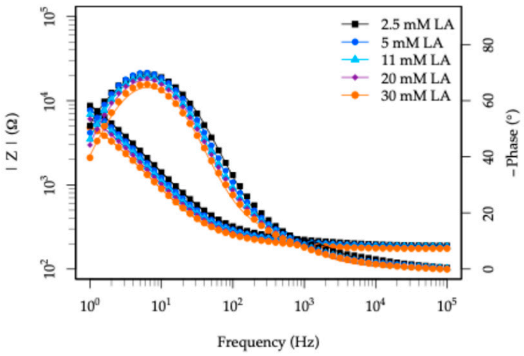

(**f**) Bode 3×TiO2 | ITO (HFE), UV

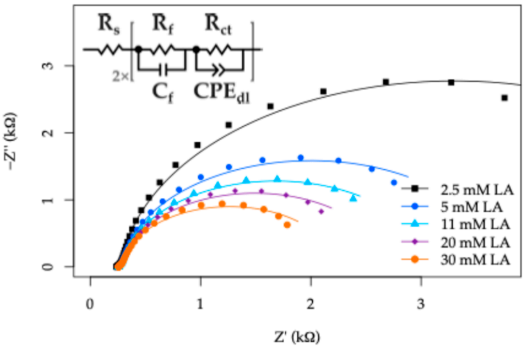

(**g**) Nyquist 5×TiO2 | ITO (HFE), UV

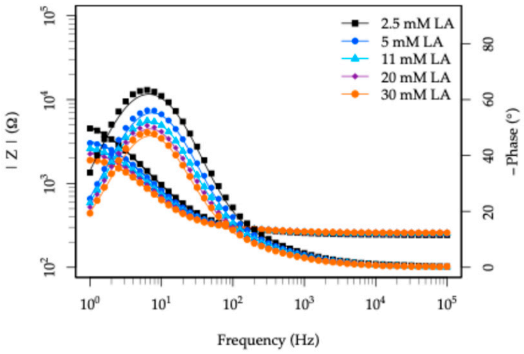

(**h**) Bode 5×TiO2 | ITO (HFE), UV

Figure S9. EIS data and fits for the Lactic acid (LA) response of the Hilbert fractal electrode (HFE) topology under UV illumination for the: (**a, b**) bare ITO; (**c, d**) 1×TiO_2 | ITO; (**e, f**) 3×TiO_2 | ITO; and (**g, h**) 5×TiO_2 | ITO.

Table S6. EIS fitting parameters, obtained for the Lactic acid (LA) response of the interdigitated electrode (IDE) and Hilbert fractal electrode (HFE) topologies under UV illumination, presented in Figures S8 & S9.

Topology	LA (mM)	R_s (Ω)	R_f (Ω)	C_f (F)	R_{ct} (Ω)	CPE_{dl}		χ^2
						(F $s^{1-\alpha}$)	α	
ITO (IDE) UV	2.5	130.2	24.0	1.17×10^{-5}	8.05×10^{6}	3.08×10^{-6}	0.97	3.00×10^{-4}
	5	131.2	34.2	1.36×10^{-5}	3.19×10^{7}	3.56×10^{-6}	0.96	5.32×10^{-4}
	11	133.4	38.0	1.39×10^{-5}	1.71×10^{13}	3.43×10^{-6}	0.96	6.60×10^{-4}
	20	133.7	56.6	1.48×10^{-5}	2.21×10^{13}	3.46×10^{-6}	0.96	1.12×10^{-3}
	30	132.1	62.1	1.55×10^{-5}	2.37×10^{13}	3.47×10^{-6}	0.97	1.12×10^{-3}
$1\times TiO_2$ I ITO (IDE) UV	2.5	134.7	15.0	1.87×10^{-5}	4.50×10^{4}	8.54×10^{-6}	0.96	1.52×10^{-4}
	5	135.9	16.0	2.38×10^{-5}	4.24×10^{4}	8.86×10^{-6}	0.96	1.03×10^{-4}
	11	137.3	16.7	2.62×10^{-5}	3.74×10^{4}	9.46×10^{-6}	0.97	8.19×10^{-5}
	20	138.1	13.4	3.17×10^{-5}	3.46×10^{4}	1.06×10^{-6}	0.97	4.50×10^{-5}
	30	137.8	10.4	4.09×10^{-5}	2.82×10^{4}	1.27×10^{-6}	0.96	3.46×10^{-5}
$3\times TiO_2$ I ITO (IDE) UV	2.5	137.8	19.1	1.50×10^{-5}	8.89×10^{3}	2.84×10^{-5}	0.92	5.77×10^{-4}
	5	139.6	26.9	3.22×10^{-5}	6.00×10^{3}	3.28×10^{-5}	0.92	2.25×10^{-4}
	11	139.3	28.0	3.82×10^{-5}	4.33×10^{3}	3.88×10^{-5}	0.92	7.13×10^{-5}
	20	138.4	23.3	5.08×10^{-5}	3.37×10^{3}	4.60×10^{-5}	0.92	6.32×10^{-4}
	30	137.1	15.4	9.23×10^{-5}	1.95×10^{3}	6.21×10^{-5}	0.91	5.42×10^{-4}
$5\times TiO_2$ I ITO (IDE) UV	2.5	134.5	17.0	6.10×10^{-5}	2.82×10^{3}	4.78×10^{-5}	0.90	1.67×10^{-4}
	5	135.6	15.4	8.24×10^{-5}	1.63×10^{3}	6.46×10^{-5}	0.89	3.18×10^{-5}
	11	137.3	13.3	1.12×10^{-4}	1.29×10^{3}	7.68×10^{-5}	0.89	1.75×10^{-5}
	20	138.1	10.1	1.71×10^{-4}	1.05×10^{3}	8.70×10^{-5}	0.89	1.78×10^{-5}
	30	137.9	5.4	3.18×10^{-4}	6.69×10^{2}	1.03×10^{-4}	0.89	4.48×10^{-5}
ITO (HFE) UV	2.5	182.0	43.4	4.42×10^{-6}	1.52×10^{6}	5.36×10^{-6}	0.94	1.36×10^{-3}
	5	187.1	64.7	6.26×10^{-6}	4.65×10^{13}	5.08×10^{-6}	0.94	2.85×10^{-3}
	11	186.9	68.3	6.09×10^{-6}	2.08×10^{13}	4.60×10^{-6}	0.94	3.06×10^{-3}
	20	184.4	84.0	6.86×10^{-6}	2.61×10^{13}	4.93×10^{-6}	0.94	3.78×10^{-3}
	30	181.7	106.5	7.40×10^{-6}	2.36×10^{14}	4.93×10^{-6}	0.95	4.92×10^{-3}
$1\times TiO_2$ I ITO (HFE) UV	2.5	194.6	35.5	5.60×10^{-6}	3.79×10^{4}	1.02×10^{-5}	0.94	9.33×10^{-4}
	5	192.4	33.0	6.02×10^{-6}	3.61×10^{4}	1.02×10^{-5}	0.95	7.16×10^{-4}
	11	190.0	34.4	6.65×10^{-6}	3.46×10^{4}	1.03×10^{-5}	0.96	7.74×10^{-4}
	20	184.5	33.4	7.44×10^{-6}	3.05×10^{4}	1.10×10^{-5}	0.96	7.61×10^{-4}
	30	178.8	34.4	8.95×10^{-6}	2.46×10^{4}	1.23×10^{-5}	0.96	8.60×10^{-4}
$3\times TiO_2$ I ITO (HFE) UV	2.5	193.7	20.6	6.53×10^{-6}	9.06×10^{3}	3.53×10^{-5}	0.90	4.91×10^{-4}
	5	191.5	23.8	1.39×10^{-5}	6.93×10^{3}	3.65×10^{-5}	0.93	4.99×10^{-4}
	11	188.2	25.7	1.58×10^{-5}	5.66×10^{3}	3.92×10^{-5}	0.94	4.02×10^{-4}
	20	183.4	23.6	1.65×10^{-5}	4.88×10^{3}	4.30×10^{-5}	0.94	4.08×10^{-4}
	30	177.5	20.4	1.82×10^{-5}	3.58×10^{3}	5.09×10^{-5}	0.93	4.90×10^{-4}
$5\times TiO_2$ I ITO (HFE) UV	2.5	245.1	16.0	2.33×10^{-5}	3.03×10^{3}	4.63×10^{-5}	0.95	5.78×10^{-4}
	5	251.3	16.7	2.66×10^{-5}	1.72×10^{3}	5.51×10^{-5}	0.95	3.73×10^{-4}
	11	255.3	16.9	2.90×10^{-5}	1.39×10^{3}	6.46×10^{-5}	0.95	1.52×10^{-4}
	20	264.1	15.2	3.36×10^{-5}	1.20×10^{3}	7.13×10^{-5}	0.94	2.98×10^{-4}
	30	259.3	15.2	3.70×10^{-5}	9.74×10^{2}	7.92×10^{-5}	0.95	4.06×10^{-4}

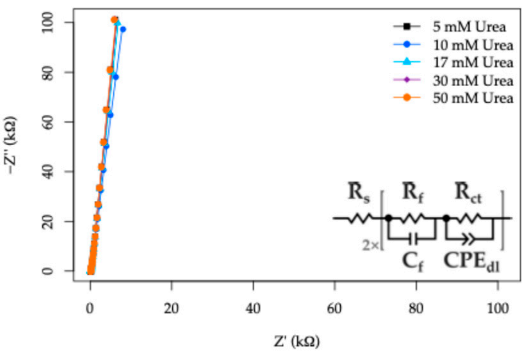

(**a**) Nyquist ITO (IDE), UV

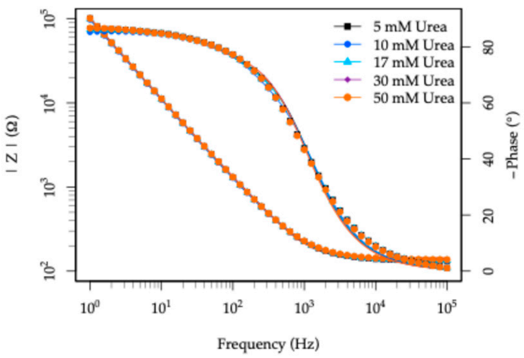

(**b**) Bode ITO (IDE), UV

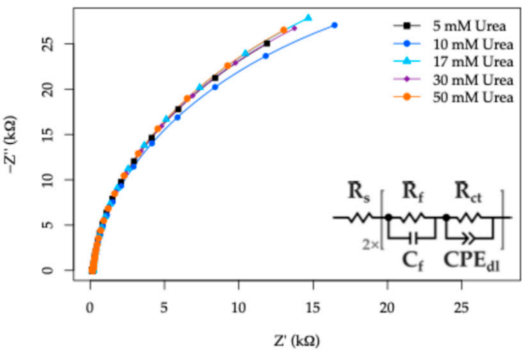

(**c**) Nyquist 1×TiO_2|ITO (IDE), UV

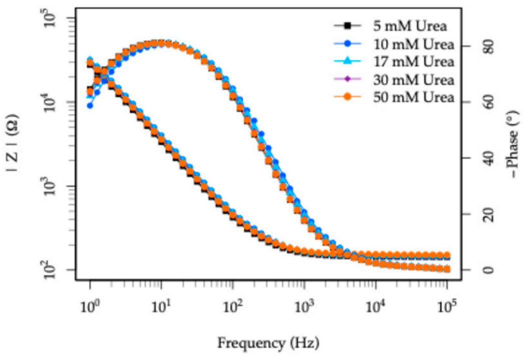

(**d**) Bode 1×TiO_2|ITO (IDE), UV

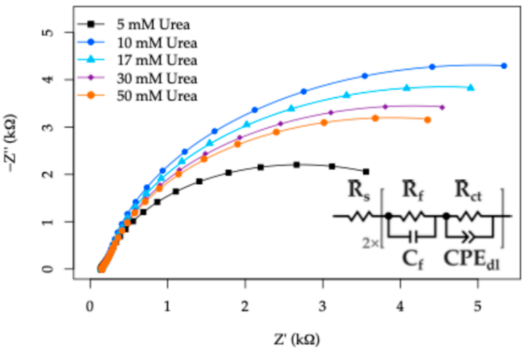

(**e**) Nyquist 3×TiO_2|ITO (IDE), UV

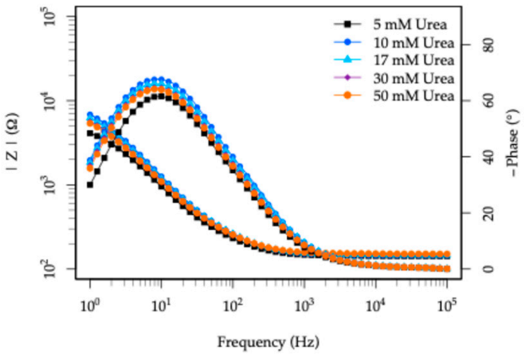

(**f**) Bode 3×TiO_2|ITO (IDE), UV

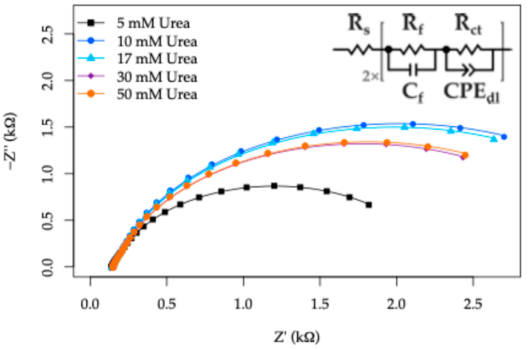

(**g**) Nyquist 5×TiO_2|ITO (IDE), UV

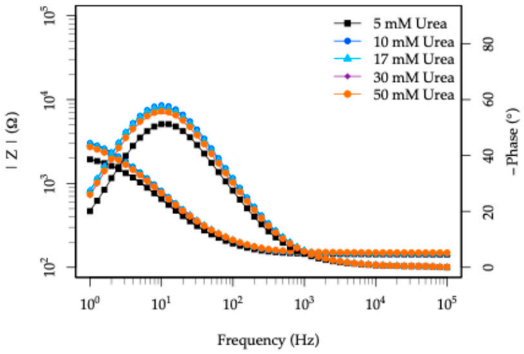

(**h**) Bode 5×TiO_2|ITO (IDE), UV

Figure S10. EIS data and fits for the Urea response of the interdigitated electrode (IDE) topology under UV illumination for the: (**a, b**) bare ITO; (**c, d**) 1×TiO_2|ITO; (**e, f**) 3×TiO_2|ITO; and (**g, h**) 5×TiO_2|ITO.

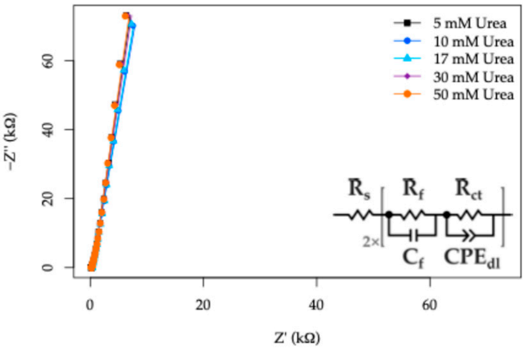

(**a**) Nyquist ITO (HFE), UV

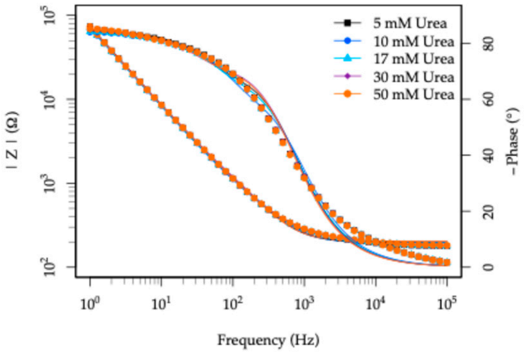

(**b**) Bode ITO (HFE), UV

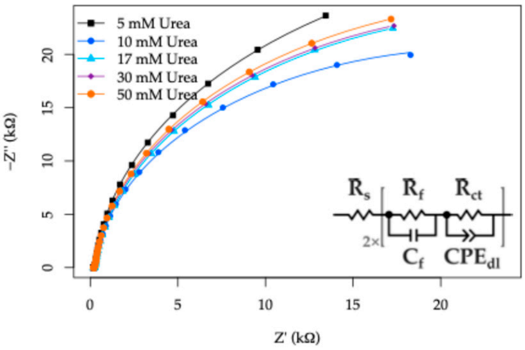

(**c**) Nyquist 1×TiO_2|ITO (HFE), UV

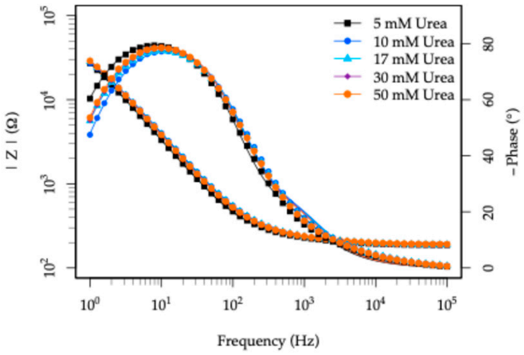

(**d**) Bode 1×TiO_2|ITO (HFE), UV

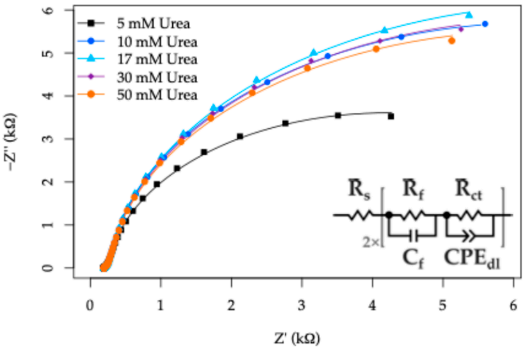

(**e**) Nyquist 3×TiO_2|ITO (HFE), UV

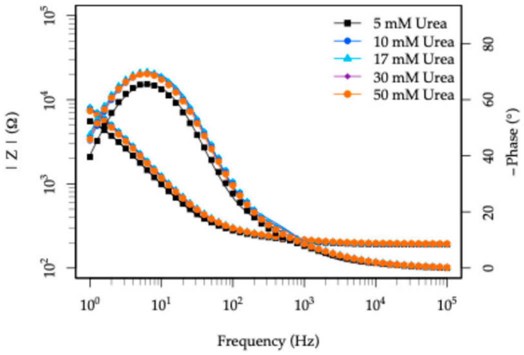

(**f**) Bode 3×TiO_2|ITO (HFE), UV

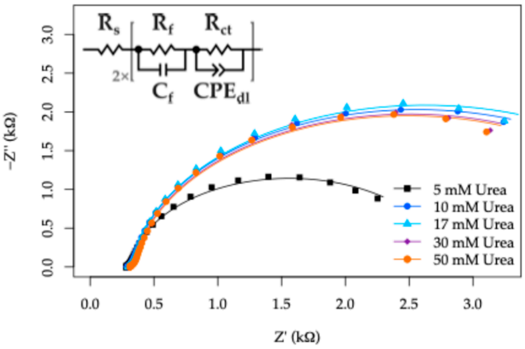

(**g**) Nyquist 5×TiO_2|ITO (HFE), UV

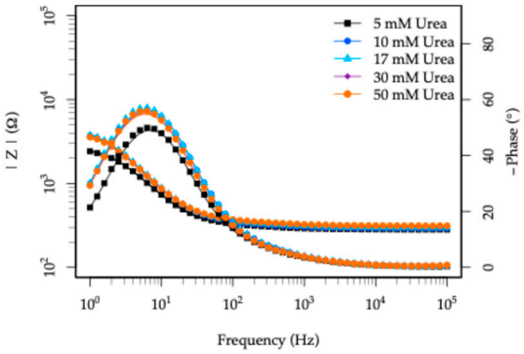

(**h**) Bode 5×TiO_2|ITO (HFE), UV

Figure S11. EIS data and fits for the Urea response of the Hilbert fractal electrode (HFE) topology under UV illumination for the: (**a, b**) bare ITO; (**c, d**) 1×TiO_2|ITO; (**e, f**) 3×TiO_2|ITO; and (**g, h**) 5×TiO_2|ITO.

Table S7. EIS fitting parameters, obtained for the Urea response of the interdigitated electrode (IDE) and Hilbert fractal electrode (HFE) topologies under UV illumination, presented in Figures S10 & S11.

**Topology**	**LA (mM)**	**R_s (Ω)**	**R_f (Ω)**	**C_f (F)**	**R_{ct} (Ω)**	**CPE_{dl}**		**χ^2**
						**(F $s^{1-\alpha}$)**	**α**	
ITO (IDE) UV	5	139.3	65.8	1.54×10^{-5}	6.11×10^{13}	3.37×10^{-6}	0.96	1.25×10^{-3}
	10	137.8	34.4	1.36×10^{-5}	1.97×10^{7}	3.56×10^{-6}	0.95	6.01×10^{-4}
	17	140.3	56.5	1.55×10^{-5}	2.85×10^{13}	3.43×10^{-6}	0.96	1.02×10^{-3}
	30	142.5	88.2	1.73×10^{-5}	9.61×10^{13}	3.36×10^{-6}	0.96	1.45×10^{-3}
	50	143.7	86.9	1.76×10^{-5}	1.07×10^{14}	3.36×10^{-6}	0.96	1.44×10^{-3}
1×TiO_2|ITO (IDE) UV	5	144.4	16.1	3.10×10^{-5}	3.73×10^{4}	1.12×10^{-5}	0.96	5.43×10^{-5}
	10	146.4	20.8	2.53×10^{-5}	3.40×10^{4}	9.33×10^{-6}	0.96	1.02×10^{-4}
	17	148.8	17.2	2.76×10^{-5}	3.85×10^{4}	9.68×10^{-6}	0.96	5.33×10^{-5}
	30	151.3	16.3	2.91×10^{-5}	3.76×10^{4}	1.02×10^{-5}	0.96	5.30×10^{-5}
	50	152.7	16.6	2.98×10^{-5}	3.87×10^{4}	1.04×10^{-5}	0.96	5.63×10^{-5}
3×TiO_2|ITO (IDE) UV	5	143.1	25.9	5.07×10^{-5}	2.51×10^{3}	4.94×10^{-5}	0.92	3.40×10^{-5}
	10	145.9	29.5	3.88×10^{-5}	4.81×10^{3}	3.49×10^{-5}	0.93	5.84×10^{-5}
	17	147.8	30.6	3.89×10^{-5}	4.36×10^{3}	3.86×10^{-5}	0.92	6.03×10^{-5}
	30	150.0	28.8	4.22×10^{-5}	3.95×10^{3}	4.23×10^{-5}	0.91	5.17×10^{-5}
	50	152.0	28.0	4.41×10^{-5}	3.67×10^{3}	4.39×10^{-5}	0.91	4.65×10^{-5}
5×TiO_2|ITO (IDE) UV	5	143.1	10.8	1.58×10^{-4}	1.04×10^{3}	8.46×10^{-5}	0.88	6.47×10^{-6}
	10	144.6	10.3	1.33×10^{-4}	1.85×10^{3}	6.67×10^{-5}	0.88	3.55×10^{-5}
	17	146.3	10.9	1.31×10^{-4}	1.82×10^{3}	6.94×10^{-5}	0.88	4.56×10^{-5}
	30	147.5	11.1	1.35×10^{-4}	1.61×10^{3}	7.34×10^{-5}	0.88	2.94×10^{-5}
	50	149.0	9.3	1.50×10^{-4}	1.65×10^{3}	7.42×10^{-5}	0.87	5.73×10^{-5}
ITO (HFE) UV	5	197.9	126.0	1.06×10^{-5}	9.34×10^{12}	4.81×10^{-6}	0.94	5.15×10^{-3}
	10	193.8	83.0	8.22×10^{-6}	4.47×10^{14}	5.11×10^{-6}	0.93	3.53×10^{-3}
	17	198.8	111.2	1.04×10^{-5}	3.91×10^{14}	5.06×10^{-6}	0.93	4.55×10^{-3}
	30	201.8	169.0	1.17×10^{-5}	1.22×10^{14}	4.83×10^{-6}	0.94	5.89×10^{-3}
	50	203.1	161.4	1.14×10^{-5}	2.27×10^{13}	4.81×10^{-6}	0.94	5.89×10^{-3}
1×TiO_2|ITO (HFE) UV	5	192.8	32.7	7.15×10^{-5}	3.06×10^{4}	1.11×10^{-5}	0.97	6.60×10^{-4}
	10	193.9	33.8	6.20×10^{-5}	2.20×10^{4}	9.56×10^{-6}	0.96	7.41×10^{-4}
	17	196.1	33.1	6.18×10^{-5}	2.59×10^{4}	9.99×10^{-6}	0.95	7.16×10^{-4}
	30	196.9	35.3	6.74×10^{-5}	2.57×10^{4}	9.76×10^{-6}	0.96	8.12×10^{-4}
	50	197.4	34.2	6.63×10^{-5}	2.66×10^{4}	9.69×10^{-6}	0.96	7.27×10^{-4}
3×TiO_2|ITO (HFE) UV	5	193.5	26.4	1.75×10^{-5}	3.96×10^{3}	4.40×10^{-5}	0.94	4.24×10^{-4}
	10	194.4	29.2	1.50×10^{-5}	6.16×10^{3}	3.29×10^{-5}	0.95	5.21×10^{-4}
	17	196.6	26.6	1.39×10^{-5}	6.74×10^{3}	3.45×10^{-5}	0.94	4.00×10^{-4}
	30	196.7	26.6	1.43×10^{-5}	6.36×10^{3}	3.54×10^{-5}	0.94	4.35×10^{-4}
	50	197.9	26.6	1.43×10^{-5}	6.03×10^{3}	3.62×10^{-5}	0.94	4.31×10^{-4}
5×TiO_2|ITO (HFE) UV	5	284.1	16.3	3.18×10^{-5}	1.24×10^{3}	6.74×10^{-5}	0.95	1.83×10^{-4}
	10	290.9	17.3	2.28×10^{-5}	2.24×10^{3}	5.48×10^{-5}	0.94	9.91×10^{-5}
	17	299.2	17.0	2.18×10^{-5}	2.29×10^{3}	5.40×10^{-5}	0.94	2.04×10^{-4}
	30	309.3	17.5	2.28×10^{-5}	2.16×10^{3}	5.55×10^{-5}	0.94	2.15×10^{-4}
	50	314.7	17.1	2.21×10^{-5}	2.14×10^{3}	5.63×10^{-5}	0.94	2.41×10^{-4}
